# Dynamics of a jointly commensurate moiré charge density wave

**DOI:** 10.1038/s41467-026-77670-2

**Published:** 2026-09-16

**Authors:** Kyoung Hun Oh, Yifan Su, Honglie Ning, B. Q. Lv, Alfred Zong, Dong Wu, Qiaomei Liu, Gyeongbo Kang, Hyeongi Choi, Hyun-Woo J. Kim, Seunghyeok Ha, Jaehwon Kim, Suchismita Sarker, Jacob P. C. Ruff, Xiaozhe Shen, Duan Luo, Stephen Weathersby, Patrick Kramer, Xinxin Cheng, Dongsung Choi, Doron Azoury, Masataka Mogi, B. J. Kim, N. L. Wang, Hoyoung Jang, Nuh Gedik

**Affiliations:** 1 Massachusetts Institute of Technology, Department of Physics, Cambridge, Massachusetts USA; 2 Tsung-Dao Lee Institute, School of Physics and Astronomy, and Zhangjiang Institute for Advanced Study, Shanghai Jiao Tong University, Shanghai, China; 3 Department of Applied Physics, Stanford University, Stanford, California USA; 4 SIMES, SLAC National Accelerator Laboratory, Menlo Park, California USA; 5 Beijing Academy of Quantum Information Sciences, Beijing, China; 6 International Center for Quantum Materials, School of Physics, Peking University, Beijing, China; 7 Pohang Accelerator Laboratory, Pohang, Gyeongbuk Republic of Korea; 8 Department of Physics, Pohang University of Science and Technology, Pohang, Republic of Korea; 9 CHESS, Cornell University, Ithaca, New York USA; 10 SLAC National Accelerator Laboratory, Menlo Park, California USA; 11 Massachusetts Institute of Technology, Department of Electrical Engineering and Computer Science, Cambridge, Massachusetts USA; 12 Department of Applied Physics, University of Tokyo, Bunkyo-ku, Tokyo Japan

**Keywords:** Phase transitions and critical phenomena, Structure of solids and liquids, Electronic properties and materials

## Abstract

The advent of two-dimensional moiré systems has revolutionized the exploration of phenomena arising from strong correlations and nontrivial band topology. Recently, a moiré superstructure formed by two coexisting charge density waves with slightly mismatched wavevectors has been realized. These incommensurate charge density waves can collectively exhibit commensurability, forming a jointly commensurate charge density wave that provides a paradigm for controlling moiré potential and periodicity. Here, we use time-resolved diffraction to probe the light-induced dynamics of the jointly commensurate charge density wave in EuTe_4_. Our measurements distinguish the instantaneous quenching of its amplitude, as verified by time-resolved photoemission spectroscopy, from the slower evolution of phase fluctuations. Moreover, while the wavevector remains locked upon photoexcitation, the correlation length decreases exclusively perpendicular to the wavevector, revealing the formation of shear-type defects. Together, our multimodal approach reconstructs the temporal evolution of the jointly commensurate charge density wave, highlighting its robustness out of equilibrium and providing insights into the optical manipulation of moiré materials through defect control.

## Abstract (web summary)

It’s been demonstrated in EuTe4 that two coexisting incommensurate charge density waves with slightly mismatched in-plane wavevectors can exhibit joint commensuration. Here, the authors demonstrate the dynamics of this jointly commensurate charge density wave and the accompanying shear-type defects.

## Introduction

**Fig. 1:**
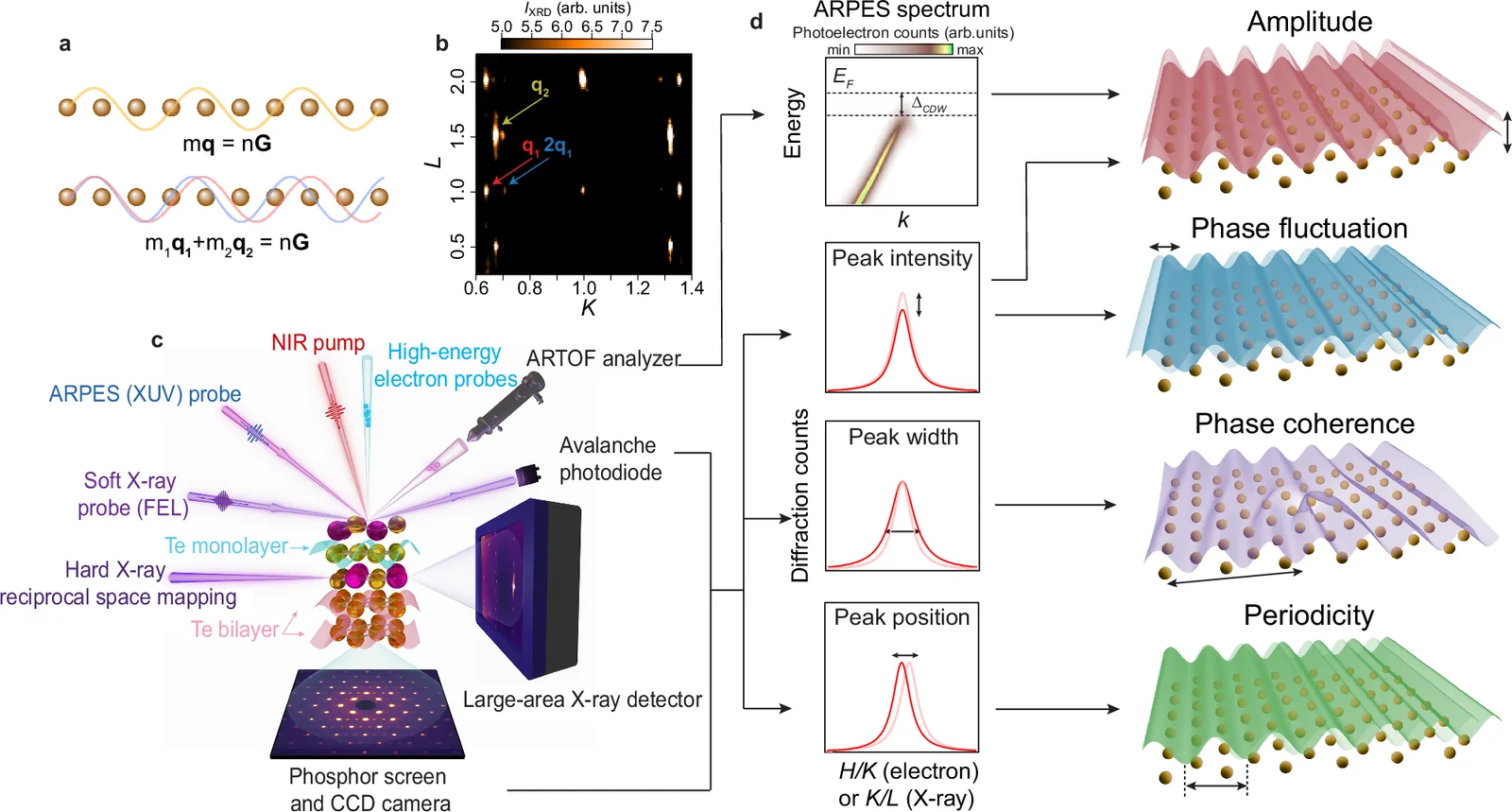
Workflow of time-and-momentum-resolved multimessenger probes for characterizing dynamics of jointly commensurate charge density wave. **a** Schematics of a commensurate CDW (top panel) and jointly commensurate CDWs (bottom panel). Spheres represent the structural lattice, and lines represent the charge density distribution. **b** Equilibrium XRD pattern in (2 *K* *L*) plane. Red, blue, and gold arrows highlight the **q**_**1**_, **2q**_**1**_ and **q**_**2**_ CDW peaks, respectively. *I*_XRD_ denotes the X-ray scattering intensity. **c** Schematic of the multi-messenger experimental setups, including static X-ray diffraction (XRD) in transmission geometry with a large-area detector, time-resolved soft X-ray diffraction (tr-XRD) in reflection geometry with an avalanche photodiode detector, time- and angle-resolved photoemission spectroscopy (tr-ARPES) with a time-of-flight detector, and ultrafast electron diffraction (UED) in transmission geometry with a phosphor screen and CCD detector. A near-infrared (NIR) pump is employed for all the time-resolved measurements. **d** Physical quantities measured by each probe. The photoemission spectrum directly measures the CDW amplitude as reflected by the CDW band gap. Diffraction intensity is associated with both CDW amplitude and phase fluctuations. Diffraction peak width specifically reflects the correlation length of the CDW order. The peak position signifies the periodicity and thus the wavevector of the CDW. Light and dark red lines denote the diffraction peak profiles before and after photoexcitation, respectively. Electron and X-ray diffraction measure the (*H* *K* 0) and (0 *K* *L*) planes, respectively.

Two-dimensional van der Waals moiré systems have opened new avenues for exploring strongly correlated and topological physics, leading to exotic phases such as correlated insulators and unconventional superconductivity^1–3^. Unlike conventional moiré systems that typically require artificial stacking, a natural, intrinsic moiré system was recently demonstrated in the newly synthesized layered material EuTe_4_, where interference and reconstruction between two charge density waves (CDWs) with slightly mismatched wavevectors produce long-wavelength modulation^4^. Here, two incommensurate charge density waves (I-CDWs) jointly lock to the lattice, forming jointly commensurate CDWs (JC-CDWs) that bridge commensurate CDWs (C-CDWs) and I-CDWs by satisfying the relation ${\sum }_{i}{m}_{i}{{{{\bf{q}}}}}_{{{{\bf{i}}}}}=n\hat{{{{\bf{G}}}}}$, where **q**_**i**_ are the CDW wavevectors of the constituent I-CDWs, $\hat{{{{\bf{G}}}}}$ is a unit reciprocal lattice vector, and *m*_*i*_, *n* are nonzero integers (Fig. 1a)^5–8^. Such a joint commensurability between coexisting CDWs has also been observed in NbSe_3_, where the relationship between the wavevectors **q**_**1**_ and **q**_**2**_ remains robust upon application of an electric current^8^.

Notably, JC-CDWs exhibit emergent properties that provide a platform for exploring fundamental physics in a charge-ordered moiré superstructure. The reconstructed long-period superlattices enable investigation of moiré phenomena in a bulk, scalable system with micron-scale coherence^4^. As the constituent I-CDWs can be responsive to perturbations, adjusting them allows engineering of the moiré potential and periodicity. Additionally, since joint commensuration is typically satisfied along a specific crystallographic direction, JC-CDWs exhibit strong electronic directionality, offering opportunities to create anisotropic spatial textures analogous to nematic or smectic electronic liquid crystal phases^9,10^. Lastly, the relative phase between individual I-CDWs introduces an additional degree of freedom, rendering an intrinsic energetic degeneracy among metastable states, which holds prospects for robust, non-volatile optical and electronic memory devices^11–15^.

Understanding and controlling these properties of JC-CDWs requires a comprehensive characterization of their response to external stimuli. Because charge orders are highly susceptible to optical excitation, their electronic states can be manipulated on ultrafast timescales, offering a pathway toward optical moiré engineering. In this context, recent studies have demonstrated photo-induced twist-angle engineering in moiré heterostructures^16^. Yet, how the spatial configuration of JC-CDWs evolves under excitation remains an open question. In particular, whether the amplitude, phase fluctuations, spatial coherence, and periodicity of each jointly locked I-CDW exhibit enhanced robustness against perturbations compared with their uncoupled counterparts remains unclear. Since this stability is closely related to the nucleation and annihilation of topological defects—features known to mediate ultrafast phase transitions in CDW systems^17–25^—it is plausible that JC-CDWs host unconventional defect dynamics, which further motivates comprehensive time-resolved characterization.

In this work, we employ a suite of time- and momentum-resolved probes to monitor the dynamics of JC-CDWs following femtosecond laser excitation^17,19–23,26–28^ to address these challenges. Leveraging a combination of time-resolved X-ray diffraction (tr-XRD), ultrafast electron diffraction (UED), and time- and angle-resolved photoemission spectroscopy (tr-ARPES), we separately track four key aspects of the CDW order parameter: amplitude, phase fluctuations, correlation length, and periodicity (Fig. 1c, d). The CDW amplitude is directly reflected in the superlattice peak intensity in time-resolved diffraction and the gap opening near the Fermi surface in tr-ARPES. The CDW phase fluctuations can be retrieved from the CDW diffraction peak intensity, analogous to the Debye-Waller effect, in which incoherent phonons quench the Bragg peak intensity^19,29^. Furthermore, the spatial phase coherence is captured by the CDW diffraction peak width, which is inversely related to the correlation length^19^. Finally, the CDW periodicity is obtained from the diffraction peak position, which directly determines the CDW wavevector. Collectively, these techniques provide a disentangled view of how different components of CDWs evolve out of equilibrium, enabling three-dimensional, real-time visualization of JC-CDW and shedding light on its ultrafast dynamics.

## Results

### Structural characterization of JC-CDW

Before studying the non-equilibrium dynamics of EuTe_4_, we first confirm the JC-CDW structure of EuTe_4_ by performing static X-ray diffraction (XRD) momentum space mapping at room temperature (See Supplementary Note 1 for full diffraction images), well below its CDW transition temperature that is above 650 K^30^. The equilibrium XRD pattern presents a set of satellite peaks at **G** ± **q**_**1**_, **G** ± **2q**_**1**_, and **G** ± **q**_**2**_, where **G** is the reciprocal lattice vector, **q**_**1**_ = (0 0.644 0), and **q**_**2**_ = (0 0.678 0.5), consistent with previous results (Fig. 1b)^11^. Previous studies suggest that the peaks at **q**_**1**_ and **q**_**2**_ originate from two competing CDWs located in the monolayer and bilayer Te sheets, respectively, and are thus referred to as the monolayer CDW and bilayer CDW^4,31^. The small in-plane mismatch between **q**_**1**_ and **q**_**2**_ results in a long-wavelength modulation, corresponding to a moiré superstructure with a periodicity of $1/{({{{{\bf{q}}}}}_{{{{\bf{1}}}}}-{{{{\bf{q}}}}}_{{{{\bf{2}}}}})}_{{{{\rm{in-plane}}}}} \sim$13.6 nm. Consistent with prior findings, the relation **q**_**1**_ + 2**q**_**2**_ = (0 2 1) holds, demonstrating that these two CDWs form a JC-CDW even at room temperature^4^. Thus, EuTe_4_ serves as an ideal testbed for investigating the properties of JC-CDWs.

### Temporal evolution of CDW order amplitude and phase fluctuations

Having established the existence of the JC-CDW in EuTe_4_ with steady-state measurements, we move on to investigate the dynamics of the CDW order parameter amplitude and phase using tr-XRD. While the intensity of a diffraction peak is often assumed to reflect only the CDW amplitude, it is also influenced by CDW phase fluctuations^19,29^. In analogy to the Debye-Waller effect from incoherent phonons that suppresses Bragg peak intensity, incoherent CDW phase fluctuations reduce the elastic CDW scattering intensity. Therefore, transient changes in diffraction intensity generally represent a convolution of amplitude suppression and enhanced phase fluctuations, making their quantitative separation challenging.

**Fig. 2:**
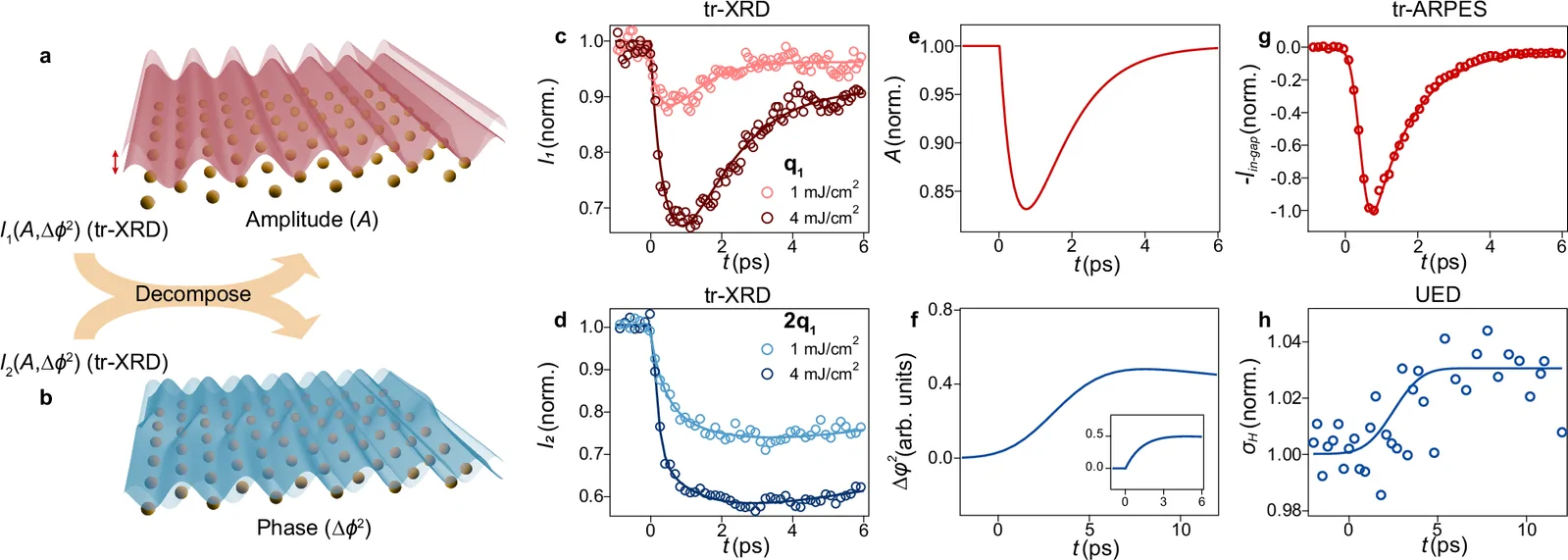
Decoding amplitude and phase fluctuations in the light-induced dynamics of the JC-CDW. **a, b** Schematics of **a** amplitude and **b** phase fluctuations. The arrow denotes the workflow to extract amplitude and phase information from first and second-order diffraction peaks in tr-XRD. **c** Temporal evolution of the intensity *I*_1_ with pump fluences *F* = 1 and 4 mJ/cm^2^ of the CDW peak at **G** ± **q**_**1**_ = (0 −0.644 1) normalized by its equilibrium value. Lines are fitted to an exponential decay. **d** Temporal evolution of the intensity *I*_2_ with pump fluences *F* = 1 and 4 mJ/cm^2^ of the CDW peak at **G** ± **2q**_**1**_ = (0 0.712 1) normalized by its equilibrium value. Lines are fitted to a bi-exponential decay. **e** Extracted temporal evolution of the monolayer CDW amplitude *A*, normalized to its equilibrium value, at a pump fluence of *F* = 4 mJ/cm^2^ from tr-XRD (Supplementary Note 2). **f** Extracted temporal evolution of the monolayer CDW phase fluctuations *Δϕ*^2^ induced by light at a pump fluence of *F* = 4 mJ/cm^2^ from tr-XRD (Supplementary Note 2). The result is further convolved with the instrumental temporal resolution of UED (~2 ps) for direct comparison with (**h**). The inset shows the original temporal evolution of *Δϕ*^2^ without accounting for the cross-correlation function. **g** Temporal evolution of the negative in-gap photoelectron spectral intensity (−*I*_*in*−*gap*_) extracted from tr-ARPES spectra taken with a pump fluence of *F* = 0.8 mJ/cm^2^, normalized to its maximal value around 0.8 ps. The solid line is a fit to a single exponential decay. **h** Temporal evolution of the diffraction peak width along the *H* direction *σ*_*H*_, defined as the statistical standard deviation of the CDW peak profile (Supplementary Note 10), with a pump fluence of *F* = 2.5 mJ/cm^2^. The solid line is a fit to a single exponential decay.

To disentangle these two effects, we develop a previously overlooked analysis scheme by tracking the dynamics of the intensity of multiple CDW peaks corresponding to the same monolayer CDW order, namely the first-order (**q**_**1**_ peak) and second-order satellite peaks (**2q**_**1**_ peak). This approach is justified as the amplitude (Fig. 2a) and phase fluctuation (Fig. 2b) modulations of the CDW contribute to changes in different satellite peak intensities to different extents^29^. Quantitatively, the intensities of the first- and second-order peaks, *I*_1_ and *I*_2_, depend on the CDW amplitude *A* and phase fluctuation *Δϕ*^2^ as

1 $${I}_{1} \sim {A}^{2}{e}^{-\frac{\Delta {\phi }^{2}}{2}}$$

2 $${I}_{2} \sim {({A}^{2}+\gamma A)}^{2}{e}^{-2\Delta {\phi }^{2}},$$

where *γ* is a constant determined by the CDW wavevector and shape (Supplementary Note 2). Therefore, this method allows us to extract the dynamics of amplitude and phase fluctuations of the target CDW by simply measuring the intensity of multiple superlattice peaks without relying on several dedicated experimental techniques.

The intensity evolution of the **q**_**1**_ and **2q**_**1**_ peaks (Fig. 2c, d) indeed exhibits stark differences: *I*_1_(*t*) rapidly decreases in ~1 ps and shows nearly full recovery within ~6 ps, while *I*_2_(*t*) slowly decreases until ~3 ps and exhibits only slight recovery up to ~6 ps. This disparity remains consistent across the investigated pump fluences from 1 to 4 mJ/cm^2^. This observation strongly suggests that the dynamics of amplitude and phase fluctuations occur on different timescales following photoexcitation. We thus leverage the aforementioned method to unambiguously disentangle the temporal evolution of both components without any a priori assumptions (Fig. 2e, f, See Supplementary Note 2 for details). The reproduced CDW amplitude decreases until ~0.8 ps and nearly fully recovers in ~6 ps (Fig. 2e). Such rapid suppression and recovery timescales are consistent with the CDW dynamics previously reported in *R*Te_3_^19^. By contrast, the extracted phase fluctuations continue to increase until ~5 ps and show only partial recovery within the time window (Fig. 2f). This result aligns with the relatively slow time scales of phase dynamics previously reported in *R*Te_3_ and nickelates^19,32^. Such observations also provide smoking-gun evidence of the significantly different formation and relaxation timescales of the amplitude and phase degrees of freedom of JC-CDWs. Compared to the rapid nonthermal dynamics of the CDW satellite peaks, the structural Bragg peaks at (0 0 1) and (0 1 2) exhibit dynamics that are slower by an order of magnitude, which can be attributed to the thermal Debye-Waller effect (Supplementary Note 3)^33–35^.

To verify the validity of the decomposition result, we employ tr-ARPES to directly track the CDW amplitude dynamics (Supplementary Note 4). As previous static ARPES experiments have demonstrated CDW gap opening in EuTe_4_^11^, we can use the in-gap spectral weight as a measure of the CDW gap size, which is proportional to the CDW amplitude^11,19,28,36–38^. As demonstrated in Fig. 2g, the negative in-gap intensity exhibits a rapid decrease up to ~0.8 ps and a nearly full recovery by ~6 ps, qualitatively resembling the temporal evolution of the monolayer CDW amplitude extracted from tr-XRD (Fig. 2e). Consequently, the tr-ARPES results successfully corroborate the validity of the CDW amplitude dynamics obtained from tr-XRD.

### Temporal evolution of CDW periodicity and correlation length

**Fig. 3:**
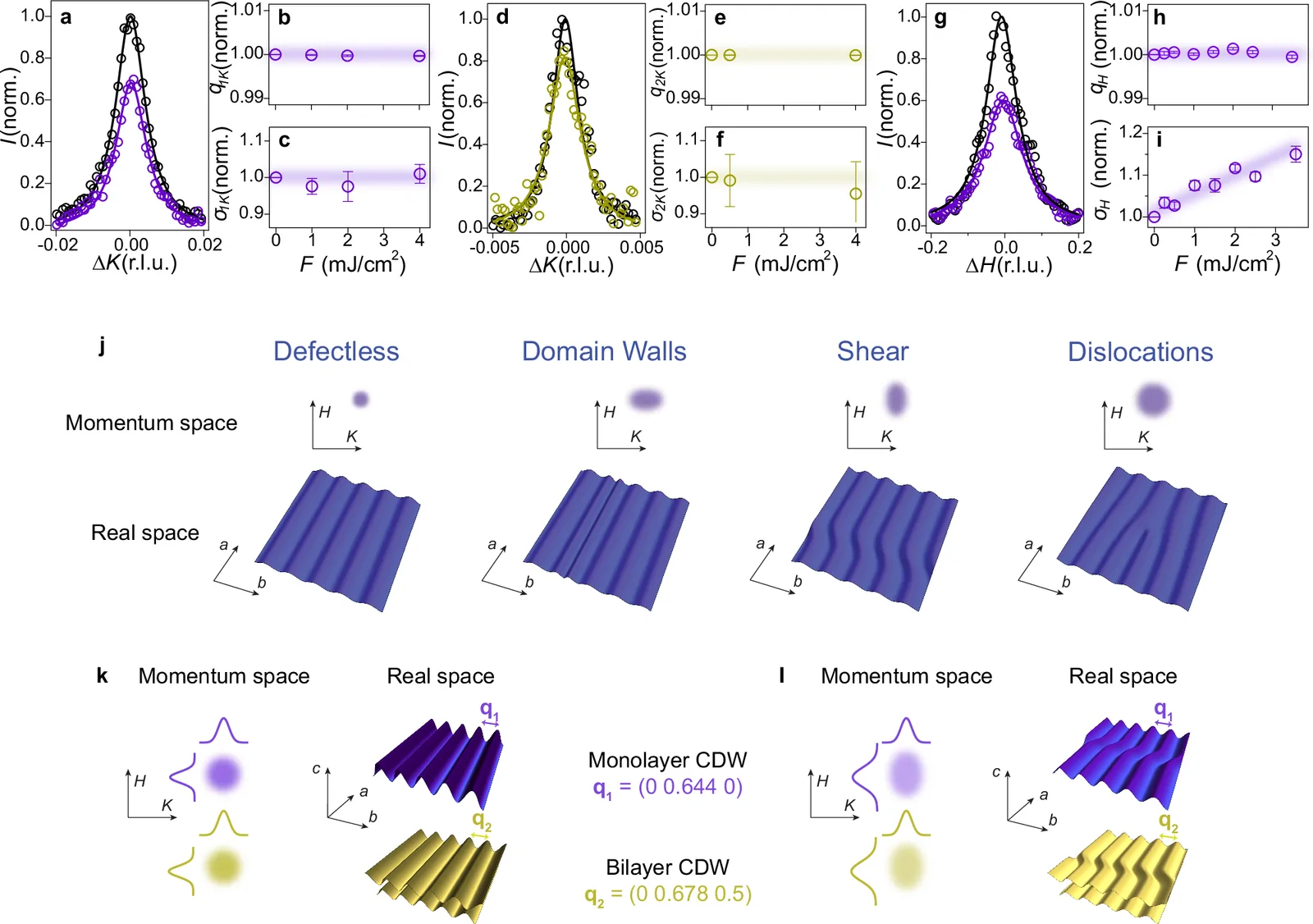
Light-induced changes in the JC-CDW periodicity and spatial coherence. **a** *K*-cut of the **q**_**1**_ peak at (0 −0.644 1) taken at *t *= −2 ps (black) and 0.5 ps (purple) with *F* = 4 mJ/cm^2^ from tr-XRD. Lines are fitted to Lorentzians. **b** Normalized position (*q*_1*K*_) and **c** width (*σ*_*K*_) defined as the full width at half maximum (FWHM) of the **q**_**1**_ peak, taken at 0.5 ps. Error bars indicate the standard deviation of the fit. Shaded lines are guides to the eyes. **d ***K*-cut of the **q**_**2**_ peak at (0 0.678 1.5) taken at *t *= −2 ps (black) and 1 ps (yellow) with *F* = 4 mJ/cm^2^ from tr-XRD. Lines are fitted to Lorentzians. **e** Normalized peak position (*q*_2*K*_) and **f** width ($\sigma_{2K}$) of the **q**_**2**_ peak, taken at 1 ps. Error bars indicate the standard deviation of the fit. Shaded lines are guides to the eyes. **g** *H*-cut of the **q**_**1**_ peak at (3 0.356 0) taken at *t *= −5 ps (black) and 4 ps (purple) with *F* = 4 mJ/cm^2^ from UED. Lines are fitted to Lorentzians. Error bars indicate the standard deviation of the fit. **h** Normalized peak position (*q*_*H*_) and **i** width (*σ*_*H*_) of the **q**_**1**_ peak along *H*, obtained from Lorentzian fitting (Supplementary Note 10), taken at 4.8 ps. Shaded lines are guides to the eyes. **j** Real and momentum space schematics of three types of CDW defects. Compared to the defect-less ground state (left panel), the three types of defects—domain walls, shear defects, and dislocations—feature longitudinal, transverse, and isotropic broadening in CDW peak widths with respect to the CDW wavevector, respectively. **k**, **l** Schematics of the **k** equilibrium and **l** excited diffraction peak shape for the monolayer CDW peak (**q**_**1**_ peak, purple) and the bilayer CDW peak (**q**_**2**_ peak, yellow). Purple (yellow) wavy patterns represent the corresponding real-space CDW configurations of the monolayer (bilayer) CDW. The intensity of the color indicates the amplitude of the order parameter. Purple (yellow) lines denote the line cuts of the CDW peaks along the *H* and *K* directions.

As we have quantitatively identified the distinct timescales of the CDW amplitude and phase fluctuation dynamics, we now turn to understanding the real-space structural evolution of the JC-CDW by tracking the CDW superlattice peak profile along the *H*, *K*, and *L* directions using time-resolved diffraction. We first examine the peak profile along the CDW wavevector direction *K* under strong photoexcitation that melts the CDW^27,39,40^. Notably, we observe no discernible change in both the monolayer (Fig. 3a, b) and bilayer (Fig. 3d, e) CDW wavevectors at the time delay when the peak intensities reach their maximal suppression. We note that this apparent invariance cannot be attributed to trivial experimental artifacts, such as pump-probe penetration-depth mismatch (Supplementary Note 5), nor to a delayed response that would emerge only at later time delays (Supplementary Note 6). This absence of peak position change reflects the persistent wavevector-locking relation between the monolayer CDW and the bilayer CDW, *q*_1*K*_ + 2*q*_2*K*_ = 2, even out of equilibrium. This behavior contrasts with typical I-CDWs, such as the dominant *c*-axis CDW in *R*Te_3_ systems that share a structural motif similar to EuTe_4_, whose wavevectors are susceptible to both temperature and light excitation^41–46^. Moreover, the peak widths along the wavevector direction of both CDWs do not broaden against light excitation either (Fig. 3c, f). This observation seemingly contradicts the peak broadening frequently observed in various CDW materials, which is attributed to the optical generation of topological defects that reduce the correlation length^19,47^. The invariant peak width upon photoexcitation indicates that the CDW defects that disrupt the correlation length along the CDW wavevector direction are not generated. The robustness of peak position and width upon excitation jointly highlights the persistence of joint commensuration even out of equilibrium, reminiscent of the stability of C-CDWs. Analogously, we also observe invariance of peak position and width along *L* (Supplementary Note 7), indicating the persistence of joint commensuration in the entire (0 *K* *L*) plane where the CDW wavevectors reside.

We then track the temporal evolution of the peak width along *H*, the other in-plane direction perpendicular to the CDW wavevectors. Although tr-XRD can unambiguously probe CDW peaks with high momentum resolution, the typical tr-XRD experimental geometry employing a two-circle diffractometer can only access peaks in the (0 *KL*) plane and prevent the detection of CDW shape changes along the *H* direction. To overcome these limitations, we utilize UED in transmission geometry, which enables time-resolved probing of peaks in the entire (*H* *K* 0) plane (Supplementary Note 8 for the full UED pattern). Since the bilayer CDW diffraction peaks are at half-integer *L* values and are therefore undetectable by UED, we henceforth focus on the monolayer CDW. We present the linecut of the representative monolayer CDW peak along *H* upon light excitation (Fig. 3g). Notably, the peak position exhibits no significant change upon photoexcitation up to 3.5 mJ/cm^2^ (Fig. 3h), reflecting the locking of CDW wavevector along *H* akin to the *K* and *L* directions. However, in contrast to them, the peak width along *H* shows a clear increase, a hallmark of the formation of light-induced defects that reduce the CDW correlation length. We note that the transverse transient broadening of the CDW diffraction peak is unlikely to originate from conventional structural disorder, in-plane hopping anisotropy of the Te 5*p* orbitals, or anisotropy in the equilibrium phase stiffness along the two in-plane directions (Supplementary Note 9). As we increase the pump fluence, the CDW peak width along *H* increases accordingly (Fig. 3i), suggesting the progressively increasing density of defects.

The exclusive peak width broadening of the monolayer CDW peak along the *H* direction indicates that light-induced CDW defects disrupt the CDW coherence selectively along *H* while preserving it along *K* and *L* (Fig. 3k, l). This behavior of the JC-CDW contrasts strikingly with both previously studied photoexcited I-CDWs, such as *R*Te_3_, and C-CDW systems, such as TiSe_2_: in *R*Te_3_, dislocation-type topological defects isotropically broaden the CDW diffraction peak along both in-plane directions^19,46^, whereas in TiSe_2_, 1D domain wall-like defects primarily broaden the CDW peak width along the wavevector direction (Fig. 3j)^22^. Our observation echoes hitherto rarely discussed shear-like defects, which form at the boundary between coherent CDW patches with a phase slip along the direction of the CDW wavevector (Fig. 3j). Therefore, these defects exclusively diminish the CDW correlation length along the in-plane direction perpendicular to the wavevector while preserving it along the wavevector (Fig. 3k, l). Consequently, our findings reveal the photo-induced formation of shear-like defects in EuTe_4_ CDWs, highlighting a distinctive nonequilibrium property of JC-CDWs. This behavior may stem from the robust wavevector-locking of the two incommensurate CDWs forming the JC-CDW, which persists even out of equilibrium. We note that previous studies have reported a phenomenologically similar effect featuring CDW wavefront bending induced by a direct current, which also results in transverse broadening of the CDW diffraction peak^48,49^. However, given the fundamental difference in generation mechanism, spatial scale, and structural requirement, such CDW bending is unlikely to be the primary origin of the anisotropic broadening observed in EuTe_4_ (Supplementary Note 9).

Last, we track the temporal evolution of the correlation length by measuring the in-plane monolayer CDW peak width along *H* with UED, the only direction that exhibits a light-induced change (Supplementary Note 10 for details). We observe a slow increase over ~4 ps and a remarkably negligible recovery within the ~12 ps time window (Fig. 2h). Importantly, this behavior qualitatively resembles the temporal evolution of the CDW phase fluctuations extracted from tr-XRD (Fig. 2f), after we take account of the order-of-magnitude lower temporal resolution of UED compared to tr-XRD. We note that CDW phase fluctuations, which are Debye-Waller-like vibrations of the electronic structure that reduce satellite peak intensity, and phase decoherence, which is the loss of spatial phase correlation that broadens the satellite peak width, are not necessarily proportional quantities during an ultrafast photoexcitation process^32^. Nevertheless, their similar temporal evolution suggests a direct connection between these two quantities in the EuTe_4_ CDW. This implies that the light-induced phase decoherence of CDW may directly result from the light-induced phase fluctuations, or vice versa.

## Discussion

**Fig. 4:**
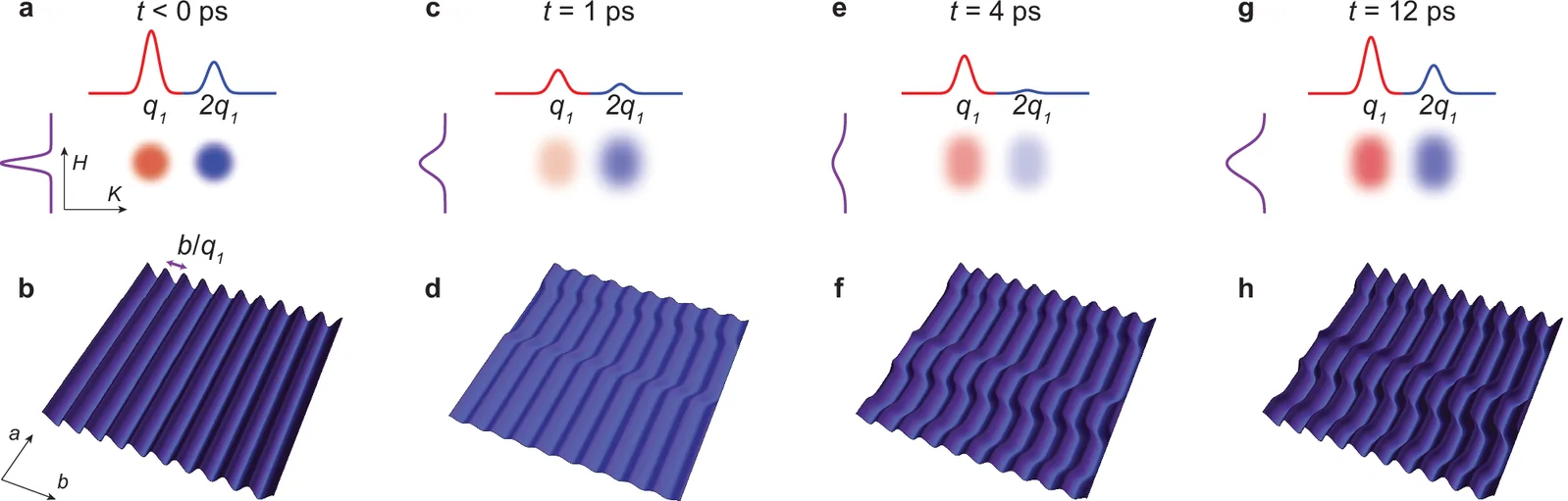
Temporal evolution of the CDW spatial configuration and shear defects. Schematics of the temporal evolution of the first- and second-order monolayer CDW peaks in momentum space and the corresponding real-space structure, at **a**, **b** *t* < 0 ps, **c**, **d** *t* = 1 ps, **e**, **f** *t* = 4 ps, and **g**, **h** *t* = 12 ps. Red (blue) lines represent the line cuts of the peak along the *K* direction at **q**_**1**_ (**2q**_**1**_). Purple lines represent the line cut of the peak along the *H* direction. Red (blue) ovals represent the shape of the **q**_**1**_ (**2q**_**1**_) satellite peaks. Purple wavy patterns represent the real-space CDW configurations of the monolayer CDW. The intensity of the color represents the amplitude of the order parameter.

With the multimodal approach combining tr-XRD, UED, and tr-ARPES, which offers momentum and time resolution, we summarize the dynamics of the monolayer CDW configuration as follows (Fig. 4a, b). Upon light irradiation, the amplitude of the monolayer CDW transiently decreases, accompanied by the formation of shear-type defects. At ~1 ps, it is maximally quenched (Fig. 4c, d), since both the in-gap spectral weight and the CDW order amplitude extracted from tr-XRD intensity show a maximal decrease. Simultaneously, the density of shear-type defects starts to grow, as indicated by the exclusive broadening of the CDW peak along the *H* direction. At4 ps, the amplitude of the monolayer CDW has mostly recovered (Fig. 4e, f), while the degree of phase fluctuations is maximized. This is accompanied by a peak in the density of shear-type defects that decreases the correlation length of the monolayer CDW. Finally, the total density of defects and CDW phase fluctuations remain nearly unchanged over ~12 ps, while the amplitude of CDW has fully recovered to equilibrium (Fig. 4g, h).

As the JC-CDW in EuTe_4_ forms a long-period moiré superstructure^4^, our observations of its light-induced dynamics show hallmarks of optical control of moiré systems. The transient suppression of the JC-CDW amplitude reflects a quenching of the moiré potential, as a reduced CDW amplitude in each layer weakens the total electrostatic potential. By contrast, the JC-CDW periodicity remains unchanged, indicating that the moiré wavelength $\sim 1/{({{{{\bf{q}}}}}_{{{{\bf{1}}}}}-{{{{\bf{q}}}}}_{{{{\bf{2}}}}})}_{{{{\rm{in-plane}}}}}$ should be preserved. This light-induced modification is distinct from conventional moiré engineering — such as varying the twist angle in twisted multilayer systems or tuning the lattice constant in lattice-mismatched heterostructures — which alter both the moiré periodicity and potential depth simultaneously. Our results demonstrate that ultrafast optical excitation can potentially suppress the depth of the moiré potential selectively while maintaining its long-range periodicity, offering an efficient route to tune moiré electronic landscapes.

In summary, with a suite of time- and momentum-resolved techniques, we investigated the ultrafast light-induced dynamics of the JC-CDW phase in EuTe_4_. Light-induced changes in the diffraction peak position and width, investigated through a combination of tr-XRD and UED, demonstrate that the periodicity and correlation length of the JC-CDW along its wavevector direction remain invariant under strong light excitation, resembling the robustness of a C-CDW rather than an I-CDW. By contrast, the correlation length of the monolayer CDW along the *H* direction is largely disrupted, signifying the ultrafast formation of shear-type defects enabled by the robustness of the wavevector-locking of the JC-CDW. On the other hand, our tr-XRD measurements unambiguously reveal the disentangled temporal evolution of the amplitude and phase fluctuations of the monolayer CDW in EuTe_4_ upon photoexcitation, achieved by simultaneously tracking the intensities of the first- and second-order diffraction peaks. Synergetic tr-ARPES and UED measurements validate this approach by revealing the distinct timescales of the melting and recovery dynamics for the CDW amplitude and phase fluctuations. The invariance of the CDW periodicity, together with its amplitude suppression, indicates that the moiré periodicity is preserved while the moiré potential depth is reduced. Our findings not only establish an approach for momentum- and time- resolved characterization of the CDW order parameter, but also provide experimental observations of JC-CDW dynamics out of equilibrium. Moreover, this dynamical methodology for distinguishing the amplitude and phase dynamics from higher-order diffraction peaks can be applied to other CDW systems where the higher-order peak intensity is strong enough. This work opens pathways not only for optical engineering of moiré systems but also for controlling CDW defects via ultrafast optical manipulation, offering prospects for uncovering emergent phases in quantum materials and enabling potential CDW-based nonvolatile memory applications^13–15,25,50^.

## Methods

### Sample preparation

EuTe_4_ single crystals were grown via a solid-phase reaction with Te as the flux. A detailed description of the synthesis process can be found elsewhere^11,12^. For synchrotron-based XRD measurements, EuTe_4_ flakes of approximately 100 nm thick were acquired by mechanical exfoliation and transferred to amorphous glass substrates. For tr-XRD measurements, EuTe_4_ bulk crystals with a dimension of approximately 2 × 1 × 0.2 mm were used. A fresh and flat surface was acquired by mechanical exfoliation before the measurement. EuTe_4_ thin flakes with a dimension of approximately 500 × 250 μm × 50 nm were mechanically exfoliated from bulk crystals and then transferred onto a copper TEM grid for the keV-UED experiments. In all measurements, the samples were mounted with their surface perpendicular to the crystalline *c*-axis. No signs of degradation were observed throughout the sample preparation and measurements. All experiments were conducted at room temperature.

### Static XRD and ED experiments

Static X-ray diffraction measurements were carried out at the Cornell High Energy Synchrotron Source (CHESS) Beamline 4B using a 29 keV beam. High-throughput volumetric reciprocal space mapping was performed in transmission mode, where the sample was rotated around an in-plane axis. The diffracted signal was collected by a Dectris Pilatus 6M detector. The ED image shown in Fig. S1b (Supplementary Note 1) is taken at the MeV-UED beamline at SLAC National Accelerator Laboratory^51^. The laser pulse was frequency-tripled to generate electron bunches from a copper photocathode. The photoelectrons were then accelerated to 4.2 MeV in a in a radio-frequency photo-injector at a repetition rate of 360 Hz. The diffraction pattern was imaged by a phosphor screen (P-43) and recorded by an electron-multiplying charge-coupled device (EMCCD, Andor iXon Ultra 888). A circular through-hole at the center of the phosphor screen allowed the undiffracted electron beam to pass through, preventing camera saturation.

### Tr-XRD experiment

All tr-XRD experiments were conducted at the SSS-RSXS endstation of PAL-XFEL^52^. The probe was a time-delayed, linearly *π*-polarized soft X-ray pulse with an 80 fs duration and a 60 Hz repetition rate. The pump was a s-polarized near-infrared (NIR) pulse, centered at 800 nm and generated by a Ti:Sapphire laser, with a 30 Hz repetition rate and pulse duration of 50 fs. The pump and probe beams were nearly collinear, with an angular separation of less than 1^∘^. The probe energy, selected to avoid resonance with the absorption edges of Eu and Te, was set to 988 eV, 1226 eV, and 1050 eV for measuring peaks at (0 −0.644 1), (0 0.678 1.5), and (0 0.712 1), respectively. The spot size of the X-ray was approximately 100 × 150 μm^2^ (FWHM) at the sample position, while the spot size of the 800 nm pump laser was approximately 500 × 500 μm (FWHM), assuming normal incidence. The X-ray scattering signal was detected using an avalanche photodiode, shielded with a 300-nm-thick aluminum filter to ensure light tightness. Data were recorded on a shot-to-shot basis using high-speed digitizers.

### UED experiment

keV-UED experiments were performed with the home-built UED system at MIT^53^. The 1038 nm (1.19 eV) output of a commercial Yb:KGW regenerative amplifier laser system (PHAROS SP-10-600-PP, Light Conversion), operating at a repetition rate of 5 kHz, was split into pump and probe branches. The pump branch was focused onto the sample for photoexcitation (s-polarization), while the probe branch was frequency quadrupled to 260 nm (4.77 eV) using two nonlinear crystals and focused onto a gold-coated sapphire in high vacuum (<3.9 × 10^−9^ torr) to generate pulsed photoelectron bunches. These electrons were accelerated to 26 keV in a DC field and focused with a solenoid onto the freestanding EuTe_4_ flakes in transmission geometry. Diffracted electrons were collected by an aluminum-coated phosphor screen (P-46), whose luminescence was recorded by a commercial intensified charge-coupled device (iCCD, PI-MAX II, Princeton Instruments) operating in the shutter mode. The nominal momentum resolution, estimated from the full width at half maximum of the raw electron beam, is ~0.03 Å^−1^. Super-resolution information down to ~1 × 10^−5^ Å^−1^ could be acquired with a regular Lorentzian fitting, as demonstrated in our previous UED experiments with the same instrument^54^. More details of the UED setup can be found elsewhere^53^.

### Tr-ARPES experiment

The 1030 nm (1.2 eV) output of either a Yb:KGW laser system (Pharos, Lightconversion) or a Yb-fiber laser system (Tangerine, Amplitude) was split into pump and probe branches, respectively. The pump branch was focused onto the cleaved surface of EuTe_4_ to excite the sample (s-polarization). The probe branch was first frequency-tripled to 344 nm (3.6 eV) and then focused onto either the hollow-core fiber filled with Xe gas or the Ar gas ejected by a gas-jet nozzle to generate the 9th harmonic at 114 nm (10.8 eV). The resulting extreme-ultraviolet (XUV) pulse was passed through a custom-built time-preserving grating monochromator (McPherson OP-XCT) to minimize pulse width broadening and enhance efficiency. After exiting the monochromator, the XUV pulse was focused onto the sample surface by a toroidal mirror. Photoelectrons were collected by a time-of-flight detector (Scienta ARTOF 10 k). More details of the tr-ARPES setup can be found elsewhere^55,56^. We note that although the data shown in the main text were acquired in the s-polarization geometry, complementary p-polarization measurements yield no discernible differences in the observed dynamics.

## Supplementary information

Supplementary information

Peer Review file

## Data Availability

The data generated in this study have been deposited in the Zenodo database https://zenodo.org/records/22149765. Supplementary datasets generated in this study are available from the corresponding author upon request.

## Code availability

The code used for simulations in this study is available from the corresponding author upon request.
