# Supplementary material for: Dynamics of a jointly commensurate moiré charge density wave
Source: Nat Commun. 2026 Sep 16;17:10893. doi: 10.1038/s41467-026-77670-2 (PMC13677753; doi:10.1038/s41467-026-77670-2)
Supplement: Supplementary file 1 [file 41467_2026_77670_MOESM1_ESM.pdf]

Supplementary Material for "Dynamics of a jointly commensurate moiré charge density wave"

Kyoung Hun Oh[1,*], Yifan Su[1,*,✉], Honglie Ning[1,*], B. Q. Lv[1,2], Alfred Zong[1,3,4], Dong Wu[5], Qiaomei Liu[6], Gyeongbo Kang[7], Hyeongi Choi[7], Hyun-Woo J. Kim[8], Seunghyeok Ha[8], Jaehwon Kim[8], Suchismita Sarker[9], Jacob P. C. Ruff[9], Xiaozhe Shen[10], Duan Luo[10], Stephen Weathersby[10], Patrick Kramer[10], Xinxin Cheng[10], Dongsung Choi[11], Doron Azoury[1], Masataka Mogi[1,12], B. J. Kim[8], N. L. Wang[5,6], Hoyoung Jang[7], and Nuh Gedik[1,✉]

[1]Department of Physics, Massachusetts Institute of Technology, Cambridge, MA 02139, USA
[2]Tsung-Dao Lee Institute, School of Physics and Astronomy, and Zhangjiang Institute for Advanced Study, Shanghai Jiao Tong University, Shanghai 200240, China
[3]Departments of Physics and of Applied Physics, Stanford University, Stanford, CA 94305, USA
[4]Stanford Institute for Materials and Energy Sciences, SLAC National Accelerator Laboratory, Menlo Park, CA 94025, USA
[5]Beijing Academy of Quantum Information Sciences, Beijing 100913, China
[6]International Center for Quantum Materials, School of Physics, Peking University, Beijing 100871, China
[7]PAL-XFEL, Pohang Accelerator Laboratory, Pohang, Gyeongbuk 37673, Republic of Korea
[8]Department of Physics, Pohang University of Science and Technology, Pohang 37673, Republic of Korea
[9]CHESS, Cornell University, Ithaca, New York 14853, USA
[10]SLAC National Accelerator Laboratory, Menlo Park, CA, USA
[11]Department of Electrical Engineering and Computer Science, Massachusetts Institute of Technology, Cambridge, MA 02139, USA
[12]Department of Applied Physics, University of Tokyo, Bunkyo-ku, Tokyo 113-8656, Japan.
✉e-mail: ys3945@columbia.edu; gedik@mit.edu

August 20, 2026

[*]These authors contributed equally to this work

Contents

Supplementary Note 1: Static XRD and ED images **3**

Supplementary Note 2: Simultaneous tracking of the amplitude and phase fluctuation dynamics **4**

Supplementary Note 3: Temporal evolution of the structural Bragg peak intensity and coherence **7**

Supplementary Note 4: CDW amplitude dynamics from tr-ARPES measurement **9**

Supplementary Note 5: Pump-probe volume mismatch in the tr-XRD experiments. **10**

Supplementary Note 6: CDW wavevector and coherence along the K direction at various time delays **11**

Supplementary Note 7: Temporal evolution of the CDW wavevector and coherence along the L direction **12**

Supplementary Note 8: Temporal evolution of the UED pattern **13**

Supplementary Note 9: Ruling out alternative explanations for the anisotropic broadening of the CDW diffraction peak **14**

Supplementary Note 10: Details of UED experimental results **17**

Supplementary Note 1: Static XRD and ED images

In this note, we present the XRD (Fig. S1a) and ED (Fig. S1b) mappings of the $(H\ K\ 0)$ planes, respectively. In both figures, a similar set of Bragg peaks and CDW satellite peaks is clearly observed. In the static ED pattern (Fig. S1b), polycrystalline rings can be seen due to imperfect crystallinity, which are excluded from the analysis. The horizontal dark crack-like pattern is due to defective detector pixels.

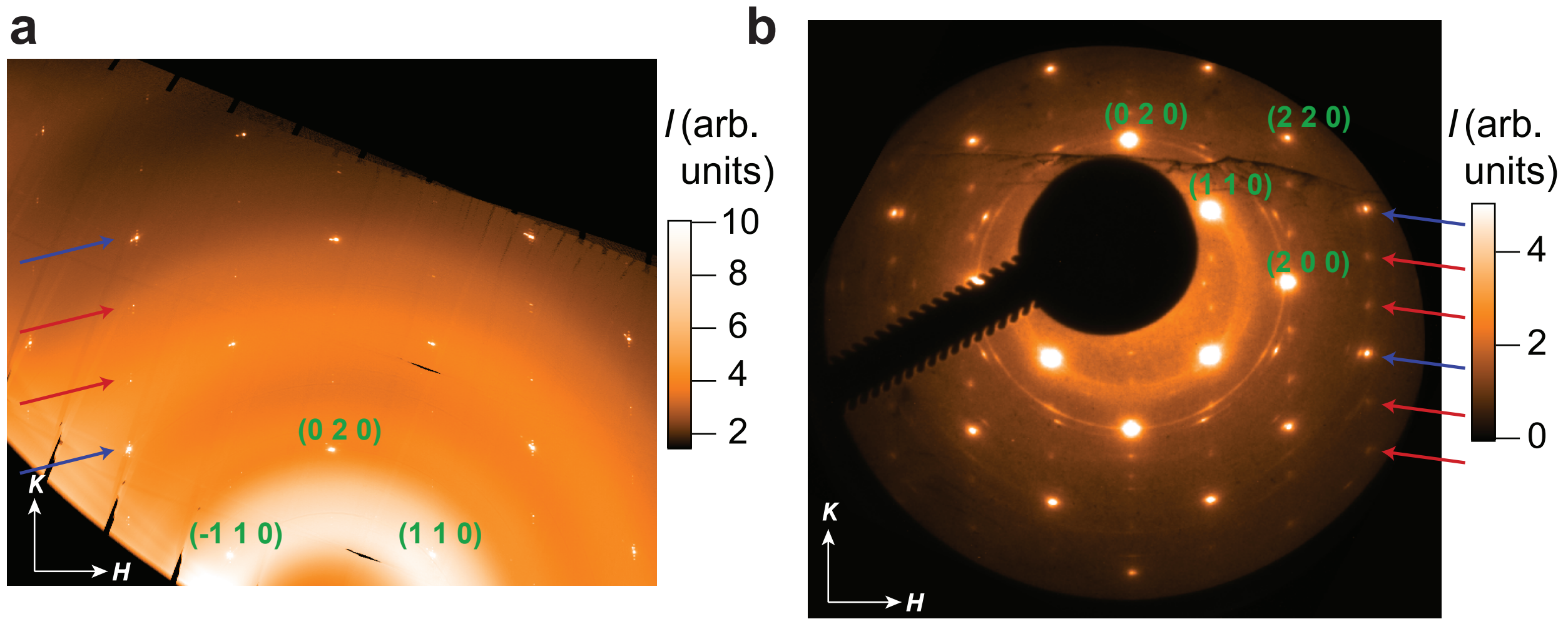

Fig. S1. **a** XRD mapping of the $(H\ K\ 0)$ plane. **b** ED mapping of the $(H\ K\ 0)$ plane. Blue and red arrows highlight structural Bragg peaks and CDW peaks, respectively.

Supplementary Note 2: Simultaneous tracking of the amplitude and phase fluctuation dynamics

In this section, we explain how the intensities of the first- and second-order CDW satellite diffraction peaks (I_1 and I_2) reveal the time scales of the amplitude and phase dynamics of the CDW.

Sinusoidal CDW

We begin by deriving expressions for the first-order and second-order satellite diffraction intensities for a perfect sinusoidal CDW distortion, which takes the form of $\vec{f}(x) = \vec{A}\sin(x)$[1]. The real-space ion density in a crystal reads:

$$\rho(\vec{r}) = \sum_{\vec{L}} \delta(\vec{r} - \vec{L} - \vec{A}\sin(\vec{q}_{\mathrm{CDW}} \cdot \vec{L})), \tag{S1}$$

where $\vec{L}$ is a lattice vector, $\vec{A}$ is a CDW distortion (amplitude) vector, and $\vec{q}_{\mathrm{CDW}}$ is a CDW wavevector. The Fourier transform of $\rho(\vec{r})$ associated with a scattering vector $\vec{K}$ is

$$\begin{aligned}\rho(\vec{K}) &= \sum_{\vec{L}} \mathrm{e}^{i\vec{K}\cdot(\vec{L}+\vec{A}\sin(\vec{q}_{\mathrm{CDW}}\cdot\vec{L}))} \\ &= \sum_{\vec{L},n} J_n(\vec{K}\cdot\vec{A}) e^{i(\vec{K}+n\vec{q}_{\mathrm{CDW}})\cdot\vec{L}}\end{aligned} \tag{S2}$$

where J_n is the nth-order Bessel of the first kind and n is an integer. Therefore, the nonzero diffraction intensity at $\vec{K} = \vec{G} - n\vec{q}_{\mathrm{CDW}}$ is

$$I(\vec{K}) \propto |J_n(\vec{K}\cdot\vec{A})|^2. \tag{S3}$$

Therefore, the expressions for the diffraction intensities for $n = 1, 2$ can be obtained. So far, the contribution from phase fluctuations has not been considered. Including the phase component $\phi(\vec{L}, t) = \sum_{\vec{q}} \phi_{\vec{q}} \sin(\vec{q}\cdot\vec{L} - \omega t)$, Eq. S2 can be modified as:

$$\begin{aligned}\rho(\vec{K}) &= \prod_{\vec{q}} \sum_{\vec{L},n} J_n(\vec{K}\cdot\vec{A}) e^{i(\vec{K}+n\vec{q}_{\mathrm{CDW}})\cdot\vec{L}} e^{in\phi_{\vec{q}}\sin(\vec{q}\cdot\vec{L}-\omega t)} \\ &= \prod_{\vec{q}} \sum_{\vec{L},n,m} J_n(\vec{K}\cdot\vec{A}) J_m(n\phi_{\vec{q}}) e^{i(\vec{K}+n\vec{q}_{\mathrm{CDW}}+m\vec{q})\cdot\vec{L}-im\omega t}\end{aligned} \tag{S4}$$

where m is an integer and $\vec{q}$ is a phason momentum. The intensities of the first- and second- order diffraction I_1 and I_2 correspond to the cases $n = 1, m = 0$ and $n = 2, m = 0$, respectively[1]. Employing the approximation $J_0(x) \sim \mathrm{e}^{-\frac{x^2}{4}}$, $J_1(x) \sim \frac{x}{2}$, and $J_2(x) \sim \frac{x^2}{8}$, the intensities take the form:

$$\begin{aligned}I_1 &\propto \prod_{\vec{q}} |J_1(\vec{K}\cdot\vec{A}) J_0(\phi_{\vec{q}})|^2 \propto \frac{1}{4}|\vec{A}|^2 \mathrm{e}^{-\frac{\phi^2}{2}} \\ I_2 &\propto \prod_{\vec{q}} |J_2(\vec{K}\cdot\vec{A}) J_0(2\phi_{\vec{q}})|^2 \propto \frac{1}{64}|\vec{A}|^4 \mathrm{e}^{-\frac{4\phi^2}{2}}\end{aligned} \tag{S5}$$

ϕ^2 is a summation of $\phi_{\vec{q}}^2$ over $\vec{q}$. Considering the distinct time dependence of the CDW amplitude $A(t)$ and phase fluctuation $\phi(t)^2$, it is possible that $I_1(t)$ and $I_2(t)$ exhibit distinct dynamics.

Nonsinusoidal CDW

Now we consider the case in which the CDW is non-sinusoidal, so that the CDW amplitude function takes the form of $\vec{f}(x) = \vec{A}_1(\sin(x) + \beta\sin(2x))$, considering up to the next leading-order term, where β is a constant.

Let us define $\vec{A}_2 = \beta \vec{A}_1$ so that $\vec{A}_1$ and $\vec{A}_2$ are first- and second-order CDW distortion vectors, respectively. Then the real-space ionic density is altered as:

$$\rho(\vec{r}) = \sum_{\vec{L}} \delta(\vec{r} - \vec{L} - \vec{A}_1 \sin(\vec{q}_{\mathrm{CDW}} \cdot \vec{L}) - \vec{A}_2 \sin(2\vec{q}_{\mathrm{CDW}} \cdot \vec{L})). \tag{S6}$$

The Fourier transform reads:

$$\begin{aligned} \rho(\vec{K}) &= \sum_{\vec{L}} \mathrm{e}^{i\vec{K}\cdot[\vec{L}+\vec{A}_1 \sin(\vec{q}_{\mathrm{CDW}}\cdot\vec{L})+\vec{A}_2 \sin(2\vec{q}_{\mathrm{CDW}}\cdot\vec{L})]} \\ &= \sum_{\vec{L},n_1,n_2} J_{n_1}(\vec{K}\cdot\vec{A}_1) J_{n_2}(\vec{K}\cdot\vec{A}_2) e^{i(\vec{K}+n_1\vec{q}_{\mathrm{CDW}}+2n_2\vec{q}_{\mathrm{CDW}})\cdot\vec{L}} \end{aligned} \tag{S7}$$

Now we include the the phase fluctuation component, $\phi(\vec{L}, t) = \sum_{\vec{q}} \phi_{\vec{q}} \sin(\vec{q}\cdot\vec{L} - \omega t)$. The $\rho(\vec{K})$ term reads:

$$\rho(\vec{K}) = \sum_{\vec{L}} \mathrm{e}^{i\vec{K}\cdot[\vec{L}+\vec{A}_1 \sin(\vec{q}_{\mathrm{CDW}}\cdot\vec{L}+\sum_{\vec{q}} \phi_{\vec{q}} \sin(\vec{q}\cdot\vec{L}-\omega t))+\vec{A}_2 \sin(2\vec{q}_{\mathrm{CDW}}\cdot\vec{L}+\sum_{\vec{q}} 2\phi_{\vec{q}} \sin(\vec{q}\cdot\vec{L}-\omega t)]} \tag{S8}$$

This expression can be simplified as follows:

$$\rho(\vec{K}) = \prod_{\vec{q}} \sum_{\vec{L},n_1,n_2,m} J_{n_1}(\vec{K}\cdot\vec{A}_1) J_{n_2}(\vec{K}\cdot\vec{A}_2) J_m[(n_1+2n_2)\phi_{\vec{q}}] \mathrm{e}^{i\vec{L}\cdot[\vec{K}+(n_1+2n_2)\vec{q}_{\mathrm{CDW}}+m\vec{q}]-im\omega t} \tag{S9}$$

The first-order diffraction peak intensity is determined by the $n_1 = 1$, $n_2 = 0$, $m = 0$ term, while the second-order diffraction term is determined by the addition of $n_1 = 2$, $n_2 = 0$, $m = 0$ and $n_1 = 0$, $n_2 = 1$, $m = 0$ terms. Therefore, the diffraction peak intensities are determined as:

$$\begin{aligned} I_1 &\propto |J_1(\vec{K}\cdot\vec{A}_1) J_0(\vec{K}\cdot\vec{A}_2) J_0(\phi)|^2 \\ I_2 &\propto |[J_2(\vec{K}\cdot\vec{A}_1) J_0(\vec{K}\cdot\vec{A}_2) + J_0(\vec{K}\cdot\vec{A}_1) J_1(\vec{K}\cdot\vec{A}_2)] J_0(2\phi)|^2 \end{aligned} \tag{S10}$$

We employ these expressions to retrieve the temporal evolution of A and $\Delta\phi^2$ $(= \phi(t)^2 - \phi^2(t < 0))$ from the tr-XRD traces (Figs. 3e,f in the main text). To this end, we use the following functional forms of A and $\Delta\phi^2$ as ansatze:

$$\begin{aligned} A(t) &= A_0[1 + a_1\Theta(t,0)(1 - \mathrm{e}^{-t/\tau_{1,1}})\mathrm{e}^{-t/\tau_{1,2}}] \\ \Delta\phi^2(t) &= a_2\Theta(t,0)(1 - \mathrm{e}^{-t/\tau_{2,1}})\mathrm{e}^{-t/\tau_{2,2}} \end{aligned} \tag{S11}$$

where $\tau_{1,1}$ $(\tau_{2,1})$ and $\tau_{1,2}$ $(\tau_{2,2})$ describe the transient melting (enhancement) and subsequent recovery time scales of the CDW amplitude (phase fluctuation), respectively. A_0 denotes the CDW amplitude before pumping, a_1 and a_2 quantify the degree of suppression (enhancement) of the CDW amplitude (phase fluctuation), and $\Theta(t, 0)$ is the Heaviside function. Eqs. S10, after incorporating Eqs. S11, are used to fit the tr-XRD results displayed in Figs. 3c,d of the main text, with the fitted results shown in Figs. 3e,f. The parameters used to fit the time traces taken with the pump fluence of 4 mJ/cm^2 are $\tau_{1,1} = 1.04 \pm 0.60$ ps, $\tau_{1,2} = 1.04 \pm 0.60$ ps, $\tau_{2,1} = 1.41 \pm 0.36$ ps, $\tau_{2,2} = 40.0 \pm 33.3$ ps, a_1=-0.68, a_2=0.58, A_0=1.87×10^{-4} Å, β=12.6. Although the uncertainty in $\tau_{2,2}$ is relatively large due to the limited time window of the tr-XRD measurement, it is clear that $\tau_{2,2}$ exceeds the time window. We note that the four time scales ($\tau_{1,1}$, $\tau_{1,2}$, $\tau_{2,1}$, $\tau_{2,2}$) remain relatively robust against variations in the initial parameters due to the pronounced differences in the intensity dynamics time scales of the first- and second-order peaks. On the other hand, we refrain from drawing conclusions based on the fitting results for other parameters (a_1, a_2, A_0, β) due to their relatively large uncertainties. To verify the fitting results, we plot the first- and second-order peak intensity dynamics, reconstructed from the amplitude and phase dynamics (Figs. 2e,f in the main text), in Fig. S2 as solid lines. The reconstructed plots show good quantitative agreement with the experimental data. In conclusion, this simultaneous tracking of the two peaks enables the disentanglement of CDW amplitude and phase fluctuation dynamics without requiring multiple experimental techniques.

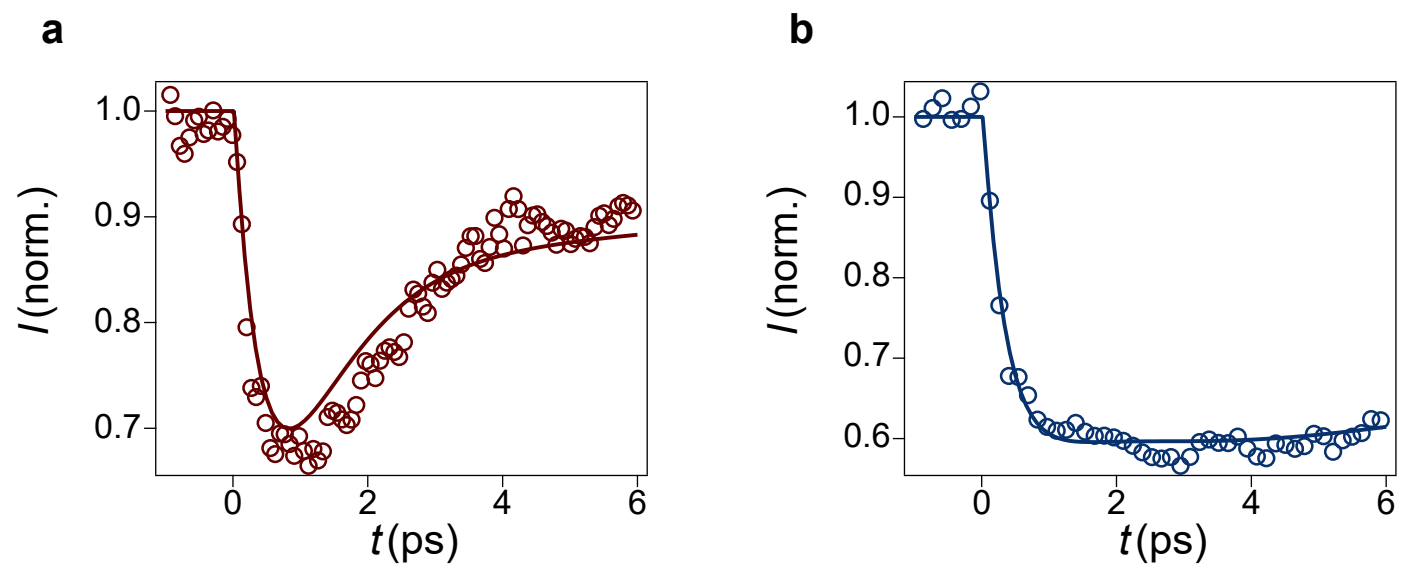

Fig. S2. **a** Temporal evolution of the intensity of the **q** peak at (0 -0.644 1) under the pump fluence of F = 4 mJ/cm^2 measured by the tr-XRD experiments. Solid line represents the first-order diffraction intensity reconstructed from the amplitude and phase dynamics. **b** Temporal evolution of the intensity of the 2**q** peak at (0 0.712 2) under the same condition. Solid line represents the second-order diffraction intensity reconstructed from the amplitude and phase dynamics. All quantities shown here are normalized with respect to their values before pumping.

Supplementary Note 3: Temporal evolution of the structural Bragg peak intensity and coherence

Here, we present the temporal evolution of structural Bragg peaks upon light excitation, measured using both tr-XRD and UED. In tr-XRD experiments, we measure two out-of-plane Bragg peaks at (0 0 1) and (0 1 2) and find that their melting is at least an order of magnitude slower than the CDW peaks, a typical timescale for the light-induced Debye-Waller effect (Figs. S3a,b). Therefore, the lattice remains nearly unchanged within the time window (< 6 ps) where CDW dynamics is observed. Similarly, the in-plane structural Bragg peaks (H K 0) measured with UED show slow melting dynamics with the intensity gradually decreasing over 15 ps (Fig. S3c). We note that the slight intensity increase within 3 ps can be attributed to the CDW-melting-induced diffraction spectral weight shift, a phenomenon commonly observed in CDW materials[2]. The intensity drop increases with fluence up to 3.5 mJ/cm^2, in contrast to the CDW peaks, whose melting saturates at $\sim$1.5 mJ/cm^2. We note that the level of Bragg peak melting measured by UED is larger than that measured with tr-XRD, possibly because UED detects peaks with higher Miller indices, which exhibit larger melting in accordance with the Debye-Waller effect.

At long time delays (>20 ps), the intensity suppression of both the Bragg and CDW peaks is affected by the Debye-Waller effect. In this regime, the relative intensity drop of the Bragg peak is approximately 50% of that for the CDW peak. This is consistent with their static temperature dependence as measured by XRD, where the relative decrease in the Bragg peak intensity is also about half of that of the CDW peak near 300 K on the heating branch[3]. We note that compared to more conventional CDW systems such as $LaTe_3$[2], the ratio of the intensity suppression of CDW satellite peaks to that of Bragg peaks is relatively small. While CDW peaks are generally expected to be more sensitive to pulsed laser excitation than lattice Bragg peaks, their quantitative difference varies substantially from material to material and from peak to peak. The relatively small response here is likely a result of the exceptional robustness of CDWs in $EuTe_4$ against photoexcitation, as shown in previous trARPES measurements[4].

In addition, the temporal evolution of the averaged structural peak width along the K and H directions is shown in Figs. S3d,e. The changes in both directions are at least an order of magnitude smaller than the CDW peak width change (Fig. 3i in the main text). This implies that the CDW peak width increase along the H direction is predominantly due to shear-type CDW defects rather than lattice defects. We also note that the structural Bragg peak width increase along the H direction is faster than that along the K direction. This suggests anisotropic formation of lattice defects, which may be related to the formation of shear-type defects in CDWs.

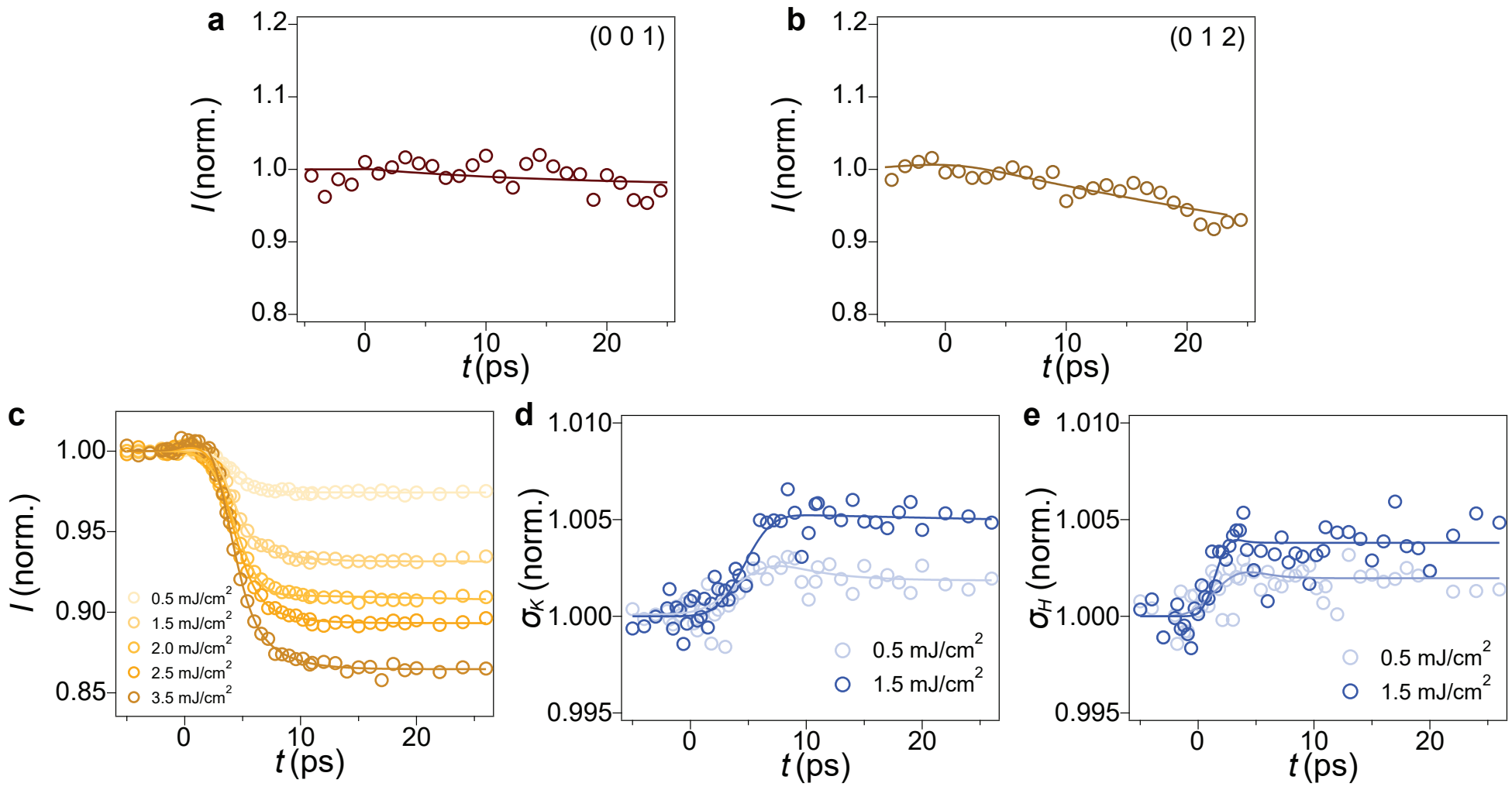

Fig. S3. Temporal evolution of the intensity of peaks at **a** (0 0 1) and **b** (0 1 2) measured by tr-XRD experiments at a fluence of 3 mJ/cm^2. Temporal evolution of **c** the averaged intensity of structural Bragg peaks, and their averaged width along **d** the K direction and **e** the H direction measured by the UED experiments. The peak widths (σ_K, σ_H) in **d,e** are the statistical standard deviations of the Bragg peak profiles along the K and H directions, respectively. Note that all quantities shown are normalized with respect to their values before pumping. Solid lines are fits to a single-exponential decay.

Supplementary Note 4: CDW amplitude dynamics from tr-ARPES measurement

In this work, we use the in-gap intensity extracted from tr-ARPES results as a measure of the monolayer CDW amplitude. Below, we provide a detailed justification for this approach. Ideally, the amplitude of the CDW order parameter should be determined by measuring the size of the CDW band gap highlighted in Fig. S4, which is attributed to the CDW formed in monolayer Te sheets. However, the asymmetry of the CDW gap around the Fermi level makes it difficult to extract the gap size beyond 1 ps, when electrons in the conduction bands relax back into the valence bands, requiring an alternative approach. Notably, as the CDW gap partially melts upon photoexcitation, the spectral weight within the gap increases. Thus, the rise in in-gap density directly reflects a decrease in the amplitude of the monolayer CDW. This approach has been validated and adopted in previous tr-ARPES measurements on $LaTe_3$ and $TbTe_3$[2,5]. We therefore use the integrated intensity within a small window near the Fermi level in the CDW gap (red rectangle in Fig. S4**a**) to represent the amplitude of the CDW. We note that the temporal evolution of the in-gap intensity is consistent with the results from gap fitting presented in previous work within the first 1 ps window[4].

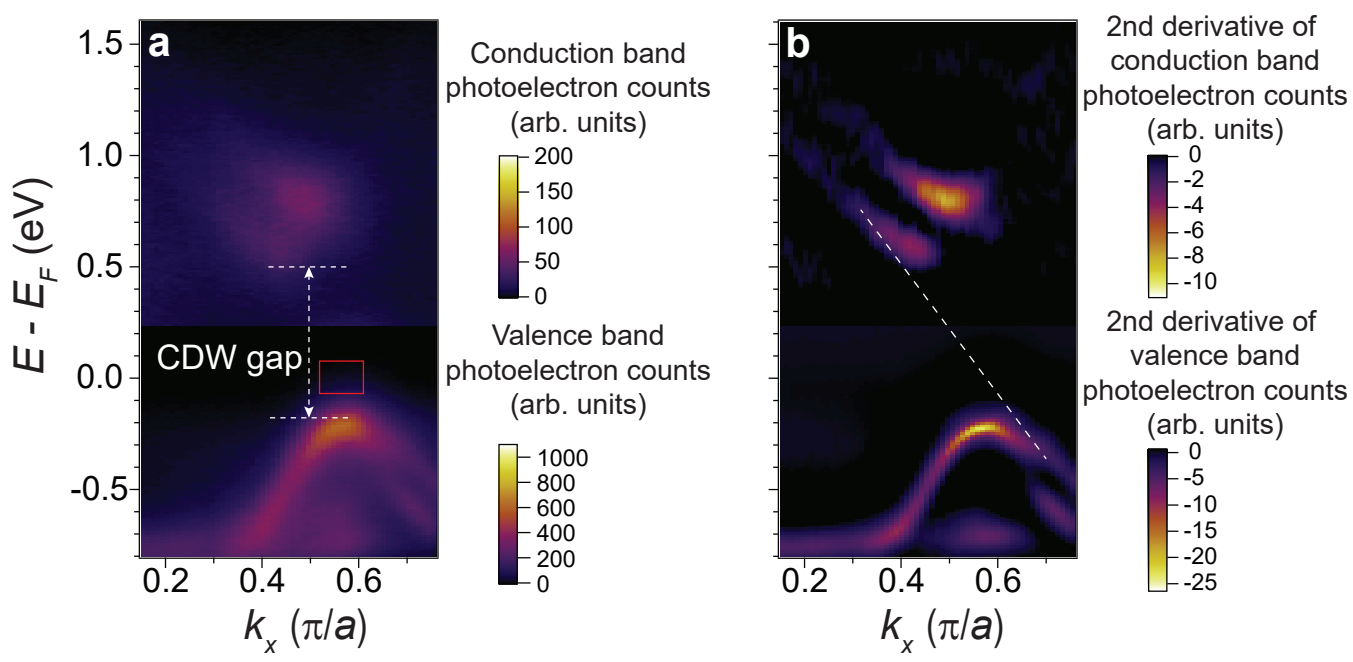

Fig. S4. a Band dispersion measured by tr-ARPES at maximum pump-probe overlap, featuring two populated conduction bands above the highlighted CDW gap. Red rectangle indicates the integration window of in-gap intensity. **b** Second derivative map of panel **a**, highlighting the band dispersion. White dashed line indicates the dispersion of the ungapped band in the normal state.

Supplementary Note 5: Pump-probe volume mismatch in the tr-XRD experiments.

In this section, we consider whether the mismatch between the optically pumped and X-ray probed volume could contribute to the observed partial suppression of the CDW diffraction intensity. Laterally, we ensured that the pump spot size was consistently ∼4 times larger than the probe spot size for all diffraction measurements, minimizing any pump-probe overlap mismatch. Regarding penetration-depth effects, although achieving perfect penetration-depth matching is challenging in the two-circle diffraction geometry in tr-XRD, the degree of mismatch is expected to be only marginal. Reported 800-nm pump penetration depths in $EuTe_4$ range from 22 to 145 nm[6,7], whereas the soft X-ray penetration depths relevant to our measurements of the monolayer and bilayer CDWs span 26 to 195 nm, depending on the photon energy and reflection geometry, suggesting only a minor pump–probe mismatch. We note that the use of soft X-rays substantially reduces the probe penetration depth and therefore mitigates pump–probe mismatch, in contrast to conventional tr-XRD experiments that typically employ hard X-rays with photon energies on the order of ∼10 keV[8].

This conclusion is further supported by our transmission UED measurements on a ∼50 nm $EuTe_4$ thin flake, performed with a 1030 nm pump and a 26 keV electron probe, where penetration-depth mismatch is minimal. The UED data (Fig. S8a) show that the intensity reduction of the monolayer CDW is comparable to that measured with soft X-rays (Fig. 2c in the main text and Fig. S2a) under similar photoexcitation conditions, confirming that penetration-depth mismatch in our X-ray measurements is indeed insignificant. Therefore, the partial CDW suppression is not an artifact of penetration-depth mismatch, and the absence of a CDW peak position shift or peak width broadening along the K direction cannot be attributed to a large pump-probe mismatch specific to the tr-XRD geometry. A similar discussion of the marginal pump–probe mismatch can be found elsewhere[9].

Supplementary Note 6: CDW wavevector and coherence along the K direction at various time delays

In this section, we show that the wavevector and coherence of the monolayer CDW along the K direction remain unchanged not only at the time delay where the CDW peak intensity is maximally quenched (Figs. 3a–c in the main text), but also at later time delays. To verify this, we performed an additional tr-XRD experiment on a different bulk $EuTe_4$ sample while keeping all other experimental conditions the same. As a reult, we observe no measurable CDW peak-position shift or peak-width broadening in the K-cut of the CDW peak at $t = 0.5$, 1, 4, and 12 ps, following photoexcitation with pump fluences of $F = 1$ and 2 mJ/cm^2 (Fig. S5). We note that the CDW peak intensity reduction at $t = 1$ ps reaches ∼16% and ∼20% for these fluences, respectively, comparable to the maximum suppression of the monolayer CDW (Fig. 2a of the main text), consistent with the saturation of CDW melting above ∼1.5 mJ/cm^2 (Supplementary Note 10).

Therefore, the absence of changes in the monolayer CDW peak ($\mathbf{q}$ peak) position and width along K cannot be attributed to insufficient optical pumping, a large pump–probe penetration-depth mismatch (Supplementary Note 5), or relatively slow temporal evolution of these quantities.

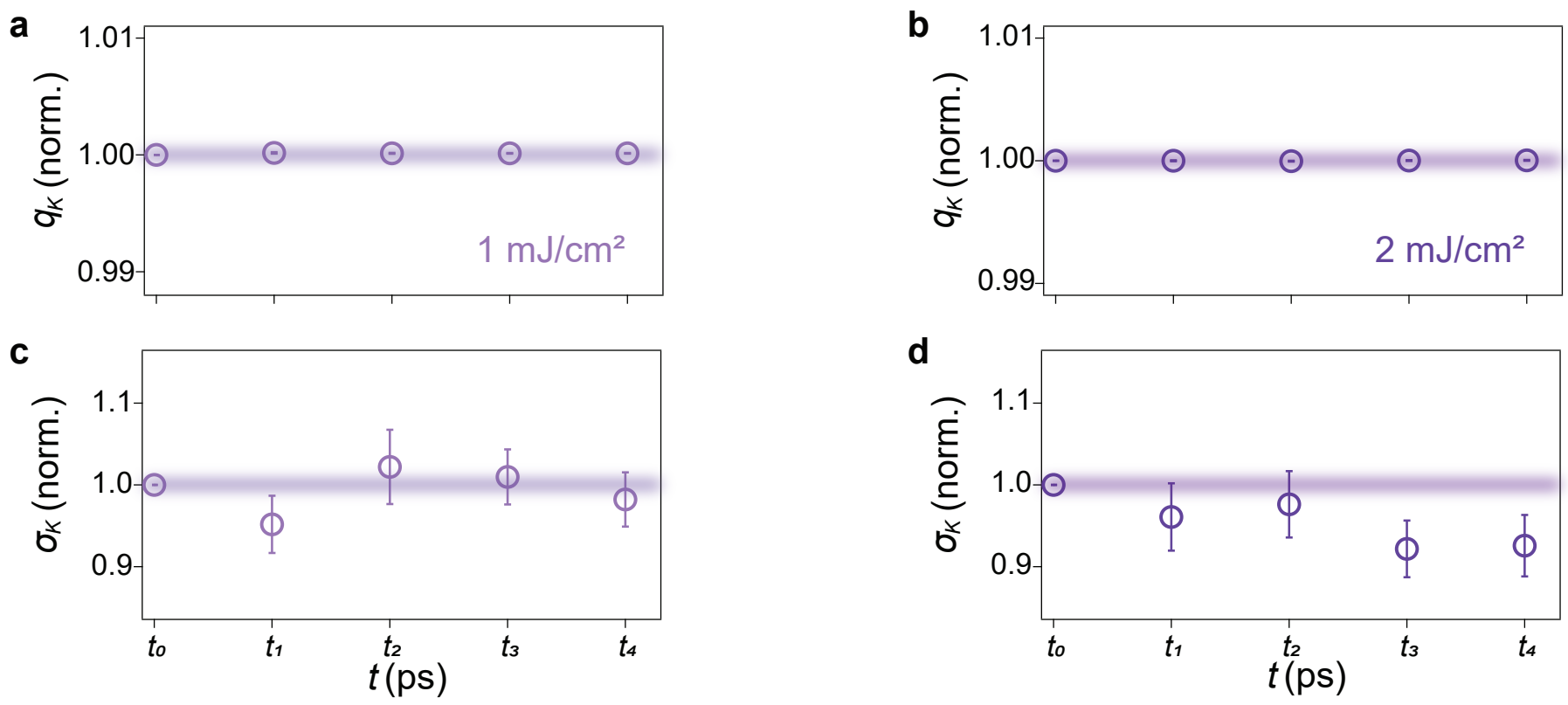

Fig. S5. Normalized position (q_K) of the $\mathbf{q}$ peak at (0 -0.644 1) with (a) $F = 1$ mJ/cm^2 and (b) $F = 2$ mJ/cm^2, taken at selected discrete time delays $t_0 < 0$, $t_1 = 0.5$, $t_2 = 1$, $t_3 = 4$, and $t_4 = 12$ (ps). Normalized width (σ_K) defined as the full width at half maximum (FWHM) of the same peak with (c) $F = 1$ mJ/cm^2 and (d) $F = 2$ mJ/cm^2, measured at the same set of delay times. Error bars indicate the standard deviation of the fit. Shaded lines are guides to the eyes.

Supplementary Note 7: Temporal evolution of the CDW wavevector and coherence along the L direction

In this work, our combined tr-XRD and UED results show that the monolayer CDW peaks at $\mathbf{G} \pm \mathbf{q}$ exhibit an intensity decrease upon photoexcitation, with negligible change in the peak position along both the H and K directions. Tr-XRD can also detect the peak position and coherence along the out-of-plane L direction, and we do not observe any signature of peak shift either (Figs. S6a,b). Therefore, the Bragg condition remains unchanged upon pumping, and the intensity decrease of the CDW peaks can be predominantly attributed to the CDW amplitude decrease. The equilibrium CDW correlation lengths, which are inversely proportional to the peak widths, are $\sim$3 unit cells along L for the monolayer CDW. As a comparison, the equilibrium CDW correlation length along K for the monolayer CDW is $>\sim$30 unit cells (Fig. 3a in the main text), highlighting the quasi-2D nature of the CDW. Similar to the K-direction, coherence along L does not exhibit a noticeable change upon light excitation, which might arise from the strong CDW-lattice locking along L.

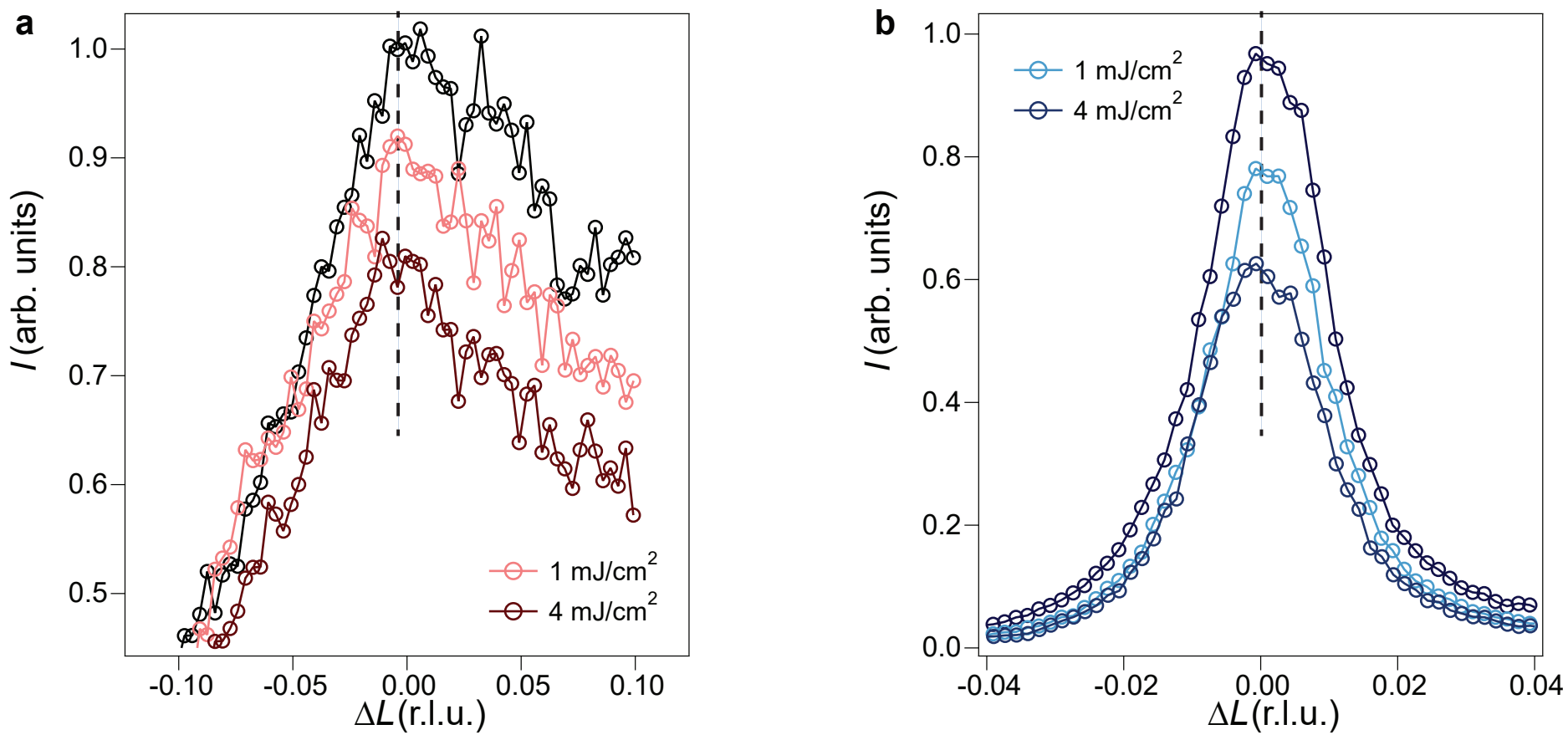

Fig. S6. L-scan cuts of the peaks at **a** (0 -0.644 1) and **b** (0 0.712 1). Black markers and lines represent the peaks in the absence of the pump, while colored markers and lines depict the peak profiles at 0.5 ps after photoexcitation. Black dashed lines serve as guides for the eyes.

Supplementary Note 8: Temporal evolution of the UED pattern

In this note, we present the temporal evolution of the ED pattern. To clearly visualize the transient changes upon light impingement, we show the ED patterns taken at several characteristic delays after pumping (Fig. S7). Each image is subtracted by the image taken before pumping ($t < 0$, Fig. S1b). In the differential ED patterns taken at $t = 1.5$ and 3.0 ps, all detected CDW peak intensities decrease, while the structural Bragg peak intensities may either increase or decrease depending on the peak indices. The different structural peak evolutions arise from the competition between CDW melting and the Debye-Waller effect: as CDW melts, the structural peak intensity is expected to increase due to the recovery of the high-temperature phase without CDWs. Simultaneously, the structural peak intensity is expected to decrease due to the ultrafast heating-induced Debye-Waller effect. After 8.0 ps, the intensity of all observed peaks decreases as the Debye-Waller effect begins to dominate.

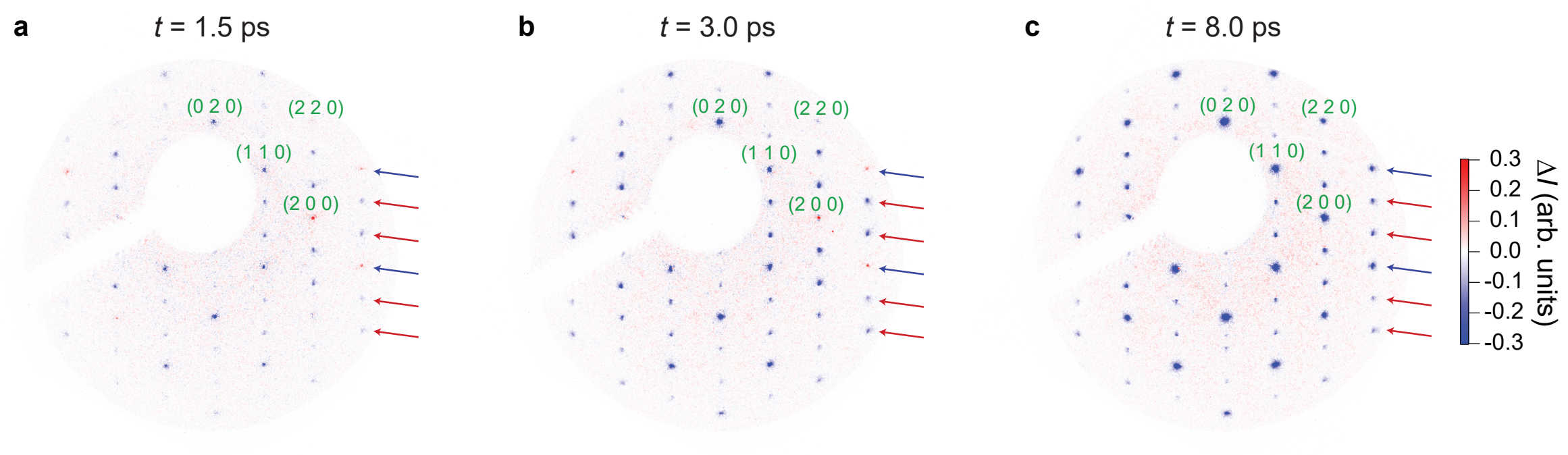

Fig. S7. Differential ED patterns (ΔI) subtracted by the ED pattern taken before pumping ($t < 0$, Fig. S1b) at selected pump-probe delays: **a** 1.5 ps, **b** 3.0 ps, and **c** 8.0 ps. Colorbar indicates ΔI in arbitrary units. Blue and red arrows highlight the Bragg and CDW peaks, respectively.

Supplementary Note 9: Ruling out alternative explanations for the anisotropic broadening of the CDW diffraction peak

In this section, we discuss the validity of an seemingly alternative scenario for the anisotropic broadening of the CDW diffraction peak in $EuTe_4$.

Structural disorder

The transverse broadening of the CDW diffraction peak cannot arise from structural disorder or conventional crystalline defects. This is based on the observation that the broadening of the CDW diffraction peaks along the H direction (Fig. S8i) is $\sim$10 times larger than that of the structural Bragg peaks measured along the same direction (Fig. S3e). The comparatively weak broadening of the structural Bragg peaks can be attributed to conventional structural disorder and defects, whereas the much larger broadening of the CDW peak indicates dominant disorder associated specifically with the CDW order.

Anisotropic hopping parameter

In tellurides such as $TbTe_3$, the hopping parameters t and t', corresponding to directions parallel and perpendicular to the in-plane Te $5p_x$ and $5p_z$ orbitals, respectively, exhibit strong anisotropy, with reported values differing by up to a factor of 5.5[10]. A similar degree of in-plane anisotropy is also expected for $EuTe_4$. Therefore, such anisotropy may appear to be related to the transverse broadening of the CDW diffraction peak observed in $EuTe_4$ upon photoexcitation. Here, however, we argue that these two phenomena are unlikely to be directly related.

First, we consider the equilibrium case. The anisotropic hopping parameters (t and t'), can potentially lead to anisotropic Fermi surface nesting. However, this does not necessarily imply that the CDW satellite diffraction peak itself is anisotropic. In fact, the anisotropy of the CDW peak width is governed by the anisotropy of the CDW phase stiffness (or equivalently the phase correlation length), which does not directly reflect differences in the hopping parameters. High-resolution XRD, ED, and STM studies on $CeTe_3$, $ErTe_3$, and $EuTe_4$ have reported no clear signatures of strong anisotropy in the CDW diffraction peaks[11–14]. This indicates that strong hopping anisotropy does not, by itself, imply strongly anisotropic CDW peak broadening in these quasi-2D tellurides.

On the other hand, for comparison, $NbSe_3$ exhibits a strong intrinsic anisotropy in the CDW phase stiffness between the chain direction and the transverse direction, as reflected in the pronounced anisotropy of the correlation lengths and CDW diffraction peak widths[15]. By contrast, $EuTe_4$ and related RTe_3 compounds are quasi-two-dimensional systems with relatively weak structural orthorhombicity and only modest anisotropy in their correlation lengths. Therefore, the transverse broadening observed in $EuTe_4$ should not be regarded as an obvious consequence of the same physics that governs quasi-1D CDWs.

We now turn to the nonequilibrium case. As discussed above, the anisotropy of the hopping parameters alone is insufficient to determine the anisotropy of the CDW diffraction peak; accordingly, any anisotropic light-induced modification of the hopping parameters is also unlikely to account for the anisotropic broadening observed here. This point is further supported by a comparison between $EuTe_4$ and RTe_3. Although these materials share similar lattice structures, anisotropic hopping parameters, and nearly isotropic equilibrium CDW peak widths, their photoinduced CDW coherence dynamics are qualitatively different. RTe_3, which hosts a single incommensurate CDW, exhibits isotropic broadening of the CDW peaks along both in-plane directions[2,16]. By contrast, $EuTe_4$, which hosts two jointly commensurate incommensurate CDWs, exhibits selective broadening of the CDW peaks primarily along the direction perpendicular to the CDW wavevector. This qualitative difference suggests that the broadening observed in $EuTe_4$ is not a generic consequence of the

underlying structural or electronic anisotropy, but is instead associated with the joint commensuration of the two incommensurate CDWs.

We further note that this broadening is unlikely to originate from simple lattice heating toward the CDW transition temperature. In both $EuTe_4$ and $LaTe_3$, the CDW transition temperature exceeds 600–700 K, whereas the pump-induced lattice heating at the maximum fluence used in this study is estimated to be only on the order of several tens of kelvin[2]. The sample therefore remains far below the transition temperature, making conventional thermal critical broadening unlikely.

Based on the above discussions, we conclude that the anisotropic broadening of the CDW peaks observed in $EuTe_4$ does not arise from hopping anisotropy, structural anisotropy, or simple lattice heating. Instead, it reflects a nonequilibrium reduction of the transverse CDW phase correlation length associated with the joint commensuration.

CDW wavefront bending effect

In recent studies, anisotropic broadening of the coherent CDW diffraction peak under an applied DC electric current, similar to our observation, was reported in $NbSe_3$[15,17]. This behavior, further confirmed by scanning X-ray microdiffraction[18], was attributed to a current-induced global bending of the CDW wavefront, caused by the pinning of the CDW at the two edges of the sample. Moreover, even in regions far from the sample edges, relatively small CDW domains or grains can give rise to similar local CDW bending effects due to pinning at domain or grain boundaries, which is conceptually closely related to the shear-type CDW defects discussed in this work. Therefore, one may speculate that photoexcitation could similarly induce bending of the CDW wavefront, leading to anisotropic broadening of the CDW diffraction peak regardless of the robustness of the joint commensuration.

However, we first note that the mechanism reported for current-induced CDW bending in $NbSe_3$ is unlikely to apply directly here. In Refs. [15, 17], CDW wavefront bending is observed under rather specific experimental conditions. In particular, $NbSe_3$ is a quasi-1D material with strong structural anisotropy, and the applied direct current is aligned parallel to the CDW wavevector. This configuration naturally favors shear-like deformation of the CDW wavefront. In contrast, $EuTe_4$ exhibits only weak in-plane structural anisotropy, and the excitation in our experiment is provided by an optical pump rather than a unidirectional DC electric current. The optical pump incoherently excites mobile carriers, which rapidly thermalize through electron–electron and electron–phonon scattering, largely erasing the initial pump polarization information. Therefore, the two key conditions underlying the CDW bending reported in the cited works—strong intrinsic lattice anisotropy and a unidirectional DC driving field—are not fulfilled in our experiment. As such, it is unlikely that the anisotropic broadening of the CDW peak observed here originates from the same mechanism as the previously reported CDW bending in $NbSe_3$.

As supporting evidence, a previous study by the same authors[19] found that the quasi-two-dimensional compound $TbTe_3$ does not exhibit clear anisotropic peak broadening under similar DC-current-driven conditions. Since $EuTe_4$ is structurally much more similar to $TbTe_3$ than to $NbSe_3$, the $NbSe_3$-type current-induced macroscopic wavefront-bending mechanism is unlikely to be responsible for the broadening observed here.

We next consider whether surface or CDW-domain-edge pinning could produce a macroscopic curvature of the CDW wavefront. This scenario is strongly constrained by the experimentally observed timescale. The CDW peak width reaches its maximum within approximately 3 ps. Taking a typical sound velocity of $\sim$2500 m/s in CDW tellurides[20], a smooth CDW deformation can propagate only over a distance of $\sim$7.5 nm within this time. It is therefore unlikely that a wavefront-curvature profile originating at the surface or domain edges could develop across an entire CDW domain (typical size $\sim$100 nm) on this timescale.

A more local version of this scenario, namely bending near pre-existing CDW grain or domain boundaries, remains possible. However, in this local limit, such curvature is phenomenologically equivalent to a shear-

type CDW defect: it locally distorts the CDW phase in the transverse direction while leaving the CDW wavelength unchanged. Since our probe beam spot size ($\sim$100 μm) is much larger than the CDW domain size ($\sim$100 nm), we cannot determine whether these shear distortions nucleate specifically at pre-existing CDW domain boundaries or at more randomly distributed positions within the pumped region. Nevertheless, both scenarios support the same conclusion that photoexcitation produces a shear-like disruption of the transverse CDW correlation length.

Importantly, even if the resulting shear-like deformation is phenomenologically similar to the CDW wavefront bending reported under DC-current-driven conditions, our observation establishes that such shear-type CDW distortions can be generated on ultrafast timescales by optical excitation, which, to the best of our knowledge, has not been reported previously. This distinguishes our work from earlier current-driven studies and highlights a nonequilibrium route for generating shear-like CDW defects through femtosecond photoexcitation.

Lastly, we note that the targeted CDW in $NbSe_3$ studied in Refs. [15, 17] is one of the two coexsiting CDWs form a jointly commensurate CDW, similar to $EuTe_4$. In $NbSe_3$, the joint commensurability relation ($2\mathbf{q_1}+2\mathbf{q_2}$=(1 1 1)) holds between the CDWs with wavevectors $\mathbf{q_1}$ and $\mathbf{q_2}$, which leads to their opposite shifts upon application of an external current well above the threshold current[21]. This behavior is consistent with the robustness of the joint commensurability upon photoexcitation demonstrated in $EuTe_4$, demonstrated in our work.

Supplementary Note 10: Details of UED experimental results

In this note, we present the UED analysis results and describe the simulation procedure used to reproduce the UED experimental results based on the tr-XRD time traces.

UED analysis details

We measured the averaged integrated intensity (Fig. S8a), position (Figs. S8b,h), and width (Figs. S8c,i) of 24 CDW peaks in the (H K 0) plane as a function of time upon light excitation (See Supplementary Note 8 for the full ED pattern at selected time delays). The peak position (Figs. S8b,h) is defined as the statistical mean position of the CDW peak profiles along either the K and H directions extracted from 24 CDW peaks, further normalized with respect to the spacing between structural Bragg peaks to compensate for thermal lattice expansion effects. The peak width (Figs. S8c,i) is defined as the statistical standard deviations of the CDW peak profiles along either the K and H directions also extracted from 24 CDW peaks. The method used to characterize peak width in this section differs from the approach applied in Fig. 3i in the main text, where Lorentzian fitting is used. This is because employing the statistical standard deviation of the CDW peak profile is computationally efficient than performing peak fitting for the simulation, a benefit we will discuss further in the following subsection. At first glance, the temporal evolution of peak position and width along the K direction from UED appears to contradict the tr-XRD results, where both quantities remain constant for the monolayer CDW. However, this discrepancy can be resolved by considering that the **q**-type and 2**q**-types peaks emerge as a single broad peak in ED due to the limited momentum resolution. In this note, we show that these observations in UED do not result from intrinsic changes in the position and width of the individual **q** and 2**q** peaks along K direction, but rather from the different degrees of melting in the intensities of these two peaks. This claim is supported by the fact that the observed timescales of the peak position and width changes (Figs. S8b,c) do not match those reported in UED experiments on Te square-net CDW systems, where the intrinsic timescales are governed by the electron–phonon coupling landscape and the phonon dispersions[2,22]. Furthermore, as will be discussed in the following subsections, we demonstrate that the temporal evolution of peak profiles can be well explained and reconciled within our modeling framework.

UED simulation details

To quantitatively simulate the temporal evolution of the peak intensity (Fig. S8a), peak position along K (Fig. S8b), and peak width along K (Fig. S8c) obtained from UED, we performed a simulation based on the tr-XRD results, further considering their momentum and temporal resolution differences. We highlight that the purpose of this simulation is not to extract physical observables from fitting, but to demonstrate how the UED results quantitatively align with the tr-XRD results. This is because the simulation accounts not only for the two targeted CDW peaks at $\mathbf{q} = (0\ 0.644\ 0)$ and $2 - 2\mathbf{q} = (0\ 0.712\ 0)$, but also for the minor CDW peaks located nearby, which increases the number of parameters involved. These minor peaks located at $4\mathbf{q} - 2 = (0\ 0.576\ 0)$ and $4 - 5\mathbf{q} = (0\ 0.780\ 0)$ correspond to the fourth and fifth order satellite peaks. Their presence is indicated by our tr-XRD line cut along (0 K 2) (Fig. S9a), where a peak around $K \sim 0.44$ can be observed, corresponding to the fourth order peak. This peak exhibits a ∼50% reduction in intensity at 2 ps after photoexcitation upon a pump of $F = 4$ mJ/cm^2, indicating its CDW origin. Although a fifth order peak at $K = \sim 0.22$ is expected to be observed, the soft x-ray wavelength in tr-XRD measurements is insufficient to reach its location. Moreover, the temporal evolution of these minor peaks was not measured in the tr-XRD experiment due to their low intensities. Therefore, for simplicity, we assume that these minor peaks approximately follow the slow dynamics of the 2**q** satellite peak. Since the intensities of the 4**q** and 5**q** peaks are relatively small compared to the dominant peak, their detailed temporal evolution only marginally affect the simulation results.

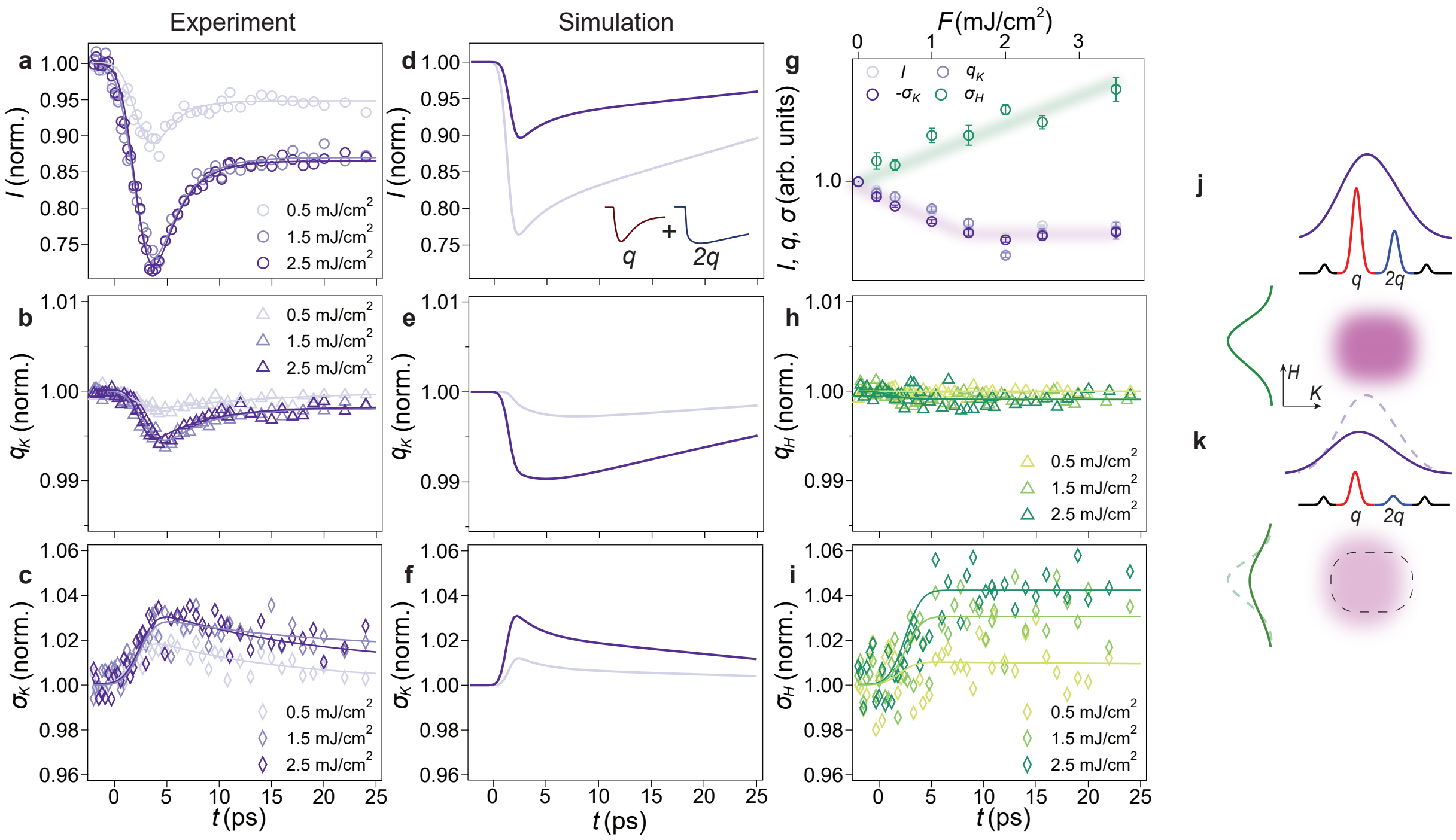

Fig. S8. Temporal evolution of the CDWs measured by UED. a-c Temporal evolution of **a** the integrated intensity I, **b** wavevector along the K direction q_K, and **c** width along the K direction σ_K, averaged among 24 CDW satellite peaks, with various pump fluences F. All data are normalized to their equilibrium values and fitted with a single exponential decay plus a constant background. **d-f**, Simulation results of the temporal evolution of **d** the peak intensity I, **e** averaged wavevector along the K direction q_K, and **f** averaged width along the K direction σ_K, with various pump fluences F (see the main text and methods for details). Insets show the temporal evolution of the **q** and 2**q** peaks from tr-XRD, which are used as the input of the simulation. **g** Pump fluence dependence of I at t = 3.9 ps, q_K at t = 4.8 ps, $-\sigma_K$ at t = 4.8 ps, and σ_H at t = 4.8 ps. Shaded purple and green lines are guides to the eyes. Error bars indicate the standard deviation of the values measured before $t = 0$. **h, i** Temporal evolution of **h** wavevector along the H direction q_H and **i** width along the H direction σ_H, averaged among 24 CDW satellite peaks, with various pump fluences F. The peak positions (q_K, q_H) in **b,h** are the statistical mean positions of the CDW peak profiles along the K and H directions, respectively, extracted from 24 CDW peaks. The positions are further normalized with respect to the spacing between structural Bragg peaks to compensate for thermal lattice expansion effects. The peak widths (σ_K, σ_H) in **c,i** are the statistical standard deviations of the CDW peak profiles along the K and H directions, respectively, also extracted from 24 CDW peaks. **j, k** Schematics of the **q** and 2**q** CDW peaks and their H- and K-cuts at $t < 0$ and t = 4 ps, respectively. Purple and green lines represent the peak line cuts obtained by UED along the K and H directions, respectively. Purple ovals represent the peak measured by UED. Red and blue lines represent the line cuts obtained by tr-XRD along the K direction for the **q** and 2**q** peaks, respectively. Black lines represent the line cuts for the weak high-order CDW peaks located nearby. Dashed lines and ovals in panel **k** depict the equilibrium values adopted from panel **j** for better comparison.

A reproduction of the UED data from our simulation, based on the tr-XRD results, can be achieved by assuming a larger static intensity for the **q** peaks and greater melting for the 2**q** peak, leading to an overall peak position shift toward lower q and an increase in the overall peak width (Figs. S8j,k). Indeed, the simulation results (Figs. S8d-f) show good qualitative agreement with the experimental UED data (Figs. S8a-c). In this simulation, we assume that the position and width of the individual **q**, 2**q**, 4**q**, 5**q** peaks do not change, as

evidenced by the tr-XRD results. We then include some free parameters that can be classified as two sets: (i) The static intensity of the $2\mathbf{q}$, $4\mathbf{q}$, $5\mathbf{q}$ peaks are defined to be $I_{2,0}$, $I_{4,0}$, $I_{5,0}$, respectively, normalized by the intensity of the $\mathbf{q}$ peak. $I_{2,0}$, $I_{4,0}$, $I_{5,0}$ are set as free parameters because their relative intensities depend on the location in reciprocal space and can vary from sample to sample. (ii) Since exact degree of pump and probe mismatch varies when measuring the CDW melting in tr-XRD and UED, the real amount of melting of the peaks at $\mathbf{q}$ and $2\mathbf{q}$ is unknown. Therefore, we introduce scaling factors α_1, α_2 to characterize the amount of the melting of these peaks. Furthermore, as mentioned above, the $4\mathbf{q}$ and $5\mathbf{q}$ peaks are assumed to follow the dynamics of the $2\mathbf{q}$ peak with the scaling factors α_4 and α_5, respectively. The temporal resolution of UED ($\sim$2 ps) is then convolved with the scaled time traces from tr-XRD to account for the temporal resolution difference. The momentum resolution of UED can be modeled as a Gaussian function with a width of 0.06, a value extracted from the width of the structural Bragg peak. This resolution function is then convolved with the widths of the four XRD peaks. Based on these inputs, the dynamics of the intensity ($I_1(t)$, $I_2(t)$, $I_4(t)$, $I_5(t)$) of the four peaks after photoexcitation are calculated. We then simulate the total intensity $I(t)$ and position $q_K(t)$ measured by UED as the sum of the intensities and the average position of the four peaks. The width $\sigma_K(t)$ measured by UED is simulated as the standard deviation of the overall peak profile, which consists of the four peaks convolved with the UED momentum resolution. We find that by assuming $I_{2,0} = 0.30$, $I_{4,0} = 0.05$, $I_{5,0} = 0.30$, $\alpha_1 = 0.23$ (or 0.60) and $\alpha_2 = 0.81$ (or 2.04), $\alpha_3 = \alpha_4 = 0.03$, a simultaneous reproduction of the experimental measured $I(t)$, $q_K(t)$, and $\sigma_K(t)$ for $F = 0.5$ mJ/cm^2 (or 1.5 mJ/cm^2) is achieved. The simulated peak profiles at selected time delays for $F = 1.5$ mJ/cm^2 clearly show the peak shift and width broadening upon light-excitation (Figs. S9b-d). We note that the combination of intensity values used in this simulation is not unique, and thus we refrain from drawing conclusions about the precise peak intensity values ($I_{2,0}$, $I_{4,0}$, and $I_{5,0}$). In summary, we find that without introducing any change in peak width or position of the individual peaks, the UED data can be quantitatively emulated, and thus the results from tr-XRD and UED are consistent.

Pump fluence dependence

Now, we examine the temporal dynamics of the CDW peaks under various pump fluences ranging from 0.25 mJ/cm^2 to 3.5 mJ/cm^2. The analysis result reveals that the peak intensity, position, and width changes along K saturate above F=1.5 mJ/cm^2 (Fig. S8a-c), and their fluence dependencies fall onto a single curve after scaled vertically (Fig. S8g), indicating an identical origin.

We note that this saturation does not arise from pump-probe misalignment or pump-induced sample damage, as the structural Bragg peaks do not exhibit such saturation in melting up to 3.5 mJ/cm^2 (Supplementary Note 3). Therefore, this result consolidates the conclusion of our UED simulation, where the varying degrees of melting across multiple peaks contribute to the changes in peak position and width observed in UED.

In contrast, the temporal evolution of the peak position and width along the H direction, the other in-plane direction perpendicular to the CDW wavevectors that is not captured by our tr-XRD measurements, exhibits significantly different dynamics and fluence dependence. Here, the peak width increases in $\sim$4 ps and persists up to $\sim$25 ps without decreasing at any pump fluences (Fig. S8i), in contrast to the much quicker recovery of the peak width along the K direction. This quasi-static value continues to increase with pump fluence without saturation, contrasting with the saturation behavior of the position and width changes along the K direction (Fig. S8i). This contrast becomes clearer if we instead quantify the peak widths along both the K and H directions (σ_K, σ_H) by fitting the peak profiles at all measured time delays, rather than using the statistical standard deviations of the CDW peak profiles as an estimate of the peak width (Figs. S8c, i). This fitting-based analysis (Fig. S10) more comprehensively visualizes the features noted above: (i) a clear contrast in the recovery time scales, with σ_K recovering about an order of magnitude faster than σ_H, and (ii) a clear saturation of the σ_K dynamics above $F = 1.5$ mJ/cm^2, as the time traces for F=1.5 – 3.5 mJ/cm^2

overlap, in contrast to σ_H. These distinct behaviors indicate that the time-dependent changes in σ_K and σ_H arise from different physical origins. Because there is no overlap of multiple CDW peaks along H, the peak broadening along H primarily reflects an intrinsic reduction of the coherence length, consistent with the generation of CDW defects that suppress coherence along H[2,23].

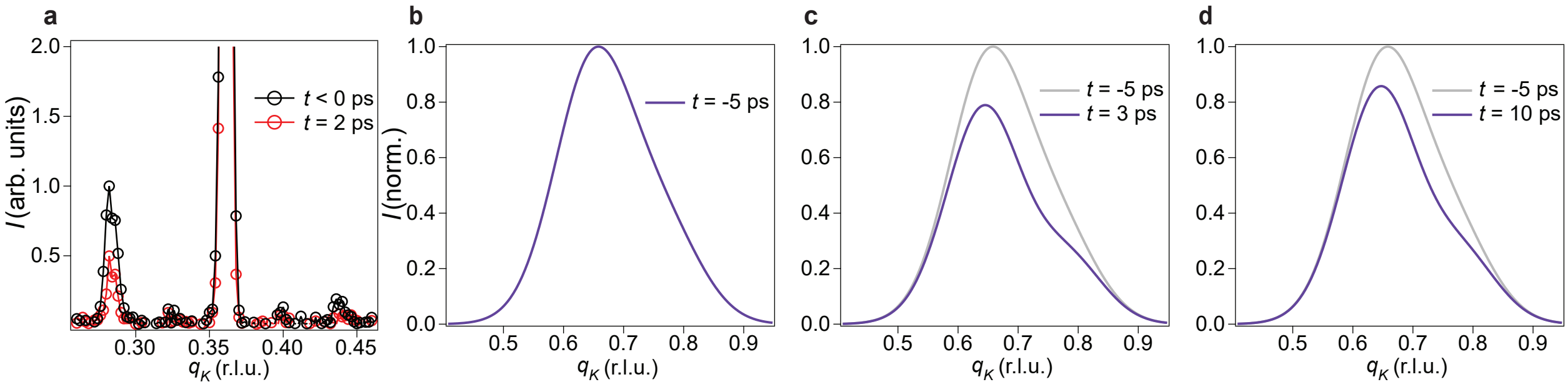

Fig. S9. **a** Tr-XRD linecuts along (0 K 2). Black markers and line depict the linecut without pumping, while red markers and line depict the linecut with a pump of 4 mJ/cm^2, taken at 2 ps. **b-d** Simulated UED peak profiles at -5 ps (before pumping), 3 ps, and 10 ps, respectively, following a 1.5 mJ/cm^2 pump. Gray curve in panels **c,d** is reproduced from panel **b** for clear comparison.

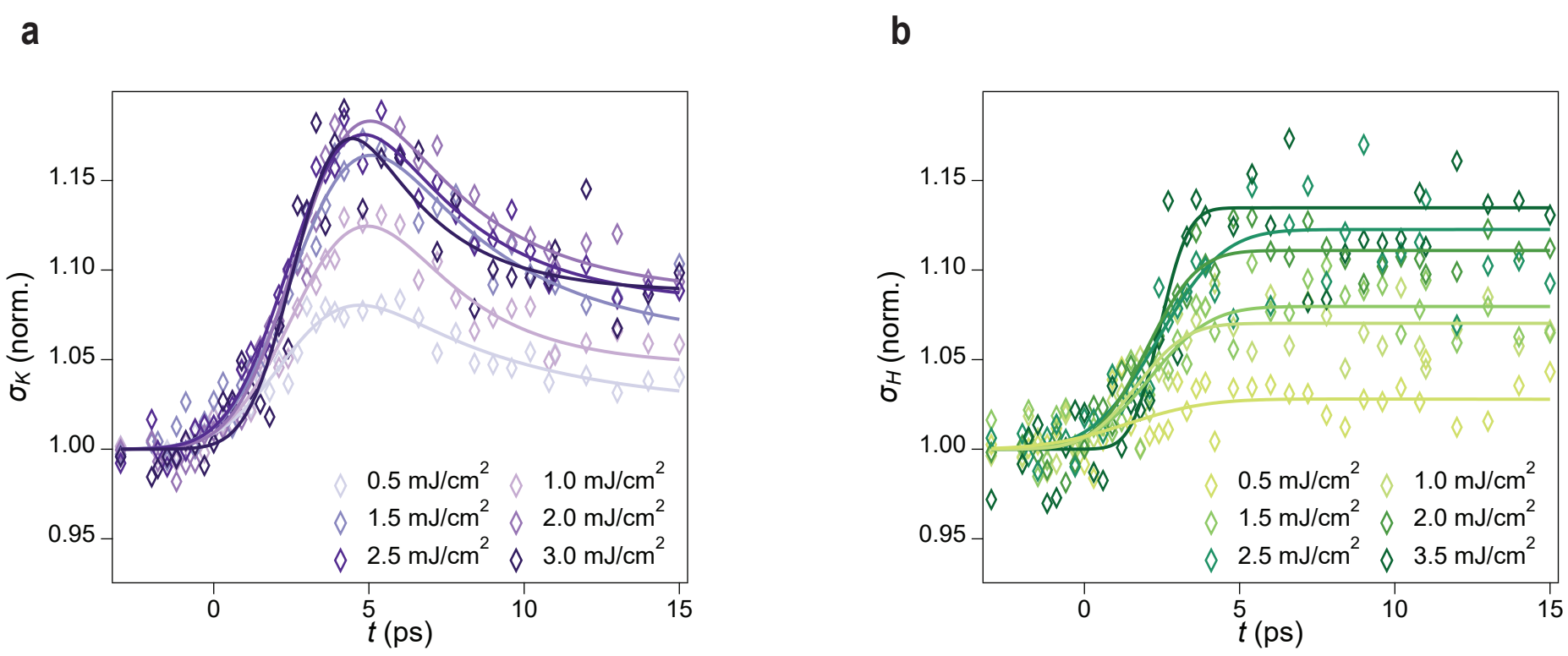

Fig. S10. Temporal evolution of the CDW peak widths along the **a** K direction σ_K and **b** H direction σ_H averaged over 24 CDW satellite peaks, with various pump fluences F, measured by UED. σ_K and σ_H are extracted by fitting the peak profiles at all measured time delays. All data are normalized to their equilibrium values and fitted with a single exponential decay plus a constant offset.

References

1. Overhauser, A. W. Observability of charge-density waves by neutron diffraction. *Phys. Rev. B* **3,** 3173–3182 (1971).
2. Zong, A. *et al.* Evidence for topological defects in a photoinduced phase transition. *Nat. Phys.* **15,** 27–31 (2019).
3. Lv, B. *et al.* Unconventional Hysteretic Transition in a Charge Density Wave. *Phys. Rev. Lett.* **128,** 036401 (2022).
4. Lv, B. *et al.* Coexistence of interacting charge density waves in a layered semiconductor. *Phys. Rev. Lett.* **132,** 206401 (2024).
5. Maklar, J. *et al.* Coherent modulation of quasiparticle scattering rates in a photoexcited charge-density-wave system. *Phys. Rev. Lett.* **128,** 026406 (2022).
6. Rathore, R. *et al.* Nonlocal Probing of Amplitude Mode Dynamics in Charge-Density-Wave Phase of $EuTe_4$. *Ultrafast Sci.* **3** (2023).
7. Liu, Q. *et al.* Room-temperature non-volatile optical manipulation of polar order in a charge density wave. *Nat. Commun.* **15,** 8937 (2024).
8. Ning, H. *et al.* Dynamical decoding of the competition between charge density waves in a kagome superconductor. *Nat. Commun.* **15,** 7286 (2024).
9. Ning, H. *et al.* Bidirectional Ultrafast Control of Charge Density Waves via Phase Competition. *Phys. Rev. Lett.* **135,** 246504 (2025).
10. Rettig, L. *et al.* Persistent order due to transiently enhanced nesting in an electronically excited charge density wave. *Nat. Commun.* **7,** 10459 (2016).
11. Okuma, R. *et al.* Highly Fluctuating Double-q Magnetic Order in the van der Waals Metal $CeTe_3$. *arXiv preprint arXiv:2604.25436* (2026).
12. Mallayya, K. *et al.* Bragg glass signatures in Pd_xErTe_3 with X-ray diffraction temperature clustering. *Nat. Phys.* **20,** 822–829 (2024).
13. Wu, D. *et al.* Layered semiconductor $EuTe_4$ with charge density wave order in square tellurium sheets. *Phys. Rev. Mater.* **3,** 024002 (2019).
14. Xiao, K. *et al.* Hidden Charge Order and Multiple Electronic Instabilities in $EuTe_4$. *Nano Lett.* (2024).
15. Pinsolle, E., Kirova, N., Jacques, V. L. R., Sinchenko, A. A. & Le Bolloc'h, D. Creep, Flow, and Phase Slippage Regimes: An Extensive View of the Sliding Charge-Density Wave Revealed by Coherent X-Ray Diffraction. *Phys. Rev. Lett.* **109,** 256402 (2012).
16. Moore, R. G. *et al.* Ultrafast resonant soft x-ray diffraction dynamics of the charge density wave in $TbTe_3$. *Phys. Rev. B* **93,** 024304 (2016).
17. Le Bolloc'h, D. *et al.* The Importance of Shear on the Collective Charge Transport in CDWs Revealed by an XFEL Source. *Sci. Adv.* **11,** eadr6034 (2025).
18. Bellec, E. *et al.* Evidence of Charge Density Wave Transverse Pinning by X-Ray Microdiffraction. *Phys. Rev. B* **101,** 125122 (2020).
19. Le Bolloc'h, D. *et al.* Effect of Dimensionality on Sliding Charge Density Waves: The Quasi-Two-Dimensional $TbTe_3$ System Probed by Coherent X-Ray Diffraction. *Phys. Rev. B* **93,** 165124 (2016).
20. Zong, A. *et al.* Role of equilibrium fluctuations in light-induced order. *Phys. Rev. Lett.* **127,** 227401 (2021).

21. Ayari, A. *et al.* Sliding-induced decoupling and charge transfer between the coexisting Q_1 and Q_2 charge density waves in $NbSe_3$. *Phys. Rev. Lett.* **93,** 106404 (2004).

22. Zhou, F. *et al.* Nonequilibrium dynamics of spontaneous symmetry breaking into a hidden state of charge-density wave. *Nat. Commun.* **12** (2021).

23. Wandel, S. *et al.* Enhanced charge density wave coherence in a light-quenched, high-temperature superconductor. *Science* **376,** 860–864 (2022).
