# Supplementary material for: Dynamics of a jointly commensurate moiré charge density wave
Source: Nat Commun. 2026 Sep 16;17:10893. doi: 10.1038/s41467-026-77670-2 (PMC13677753; doi:10.1038/s41467-026-77670-2)
Supplement: Supplementary file 2 [file 41467_2026_77670_MOESM2_ESM.pdf]

nature portfolio

Peer Review File

Dynamics of a jointly commensurate moiré charge density wave

Corresponding Author: Professor Nuh Gedik

Parts of this Peer Review File have been redacted as indicated to remove third-party material.

This file contains all reviewer reports in order by version, followed by all author rebuttals in order by version.

Version 0:

Reviewer comments:

Reviewer #1

(Remarks to the Author)
Referee Report on “Dynamics of a jointly commensurate moiré charge density wave” by Oh et al.
This manuscript reports time-resolved studies of the charge density wave (CDW) dynamics in $EuTe_4$ using a combination of ultrafast x-ray diffraction, electron diffraction, and time-resolved ARPES. The CDW in this material forms a moiré superstructure resulting from two incommensurate CDWs. The authors report that photoexcitation reduces the CDW amplitude while the diffraction peaks develop an anisotropic broadening, which they interpret as evidence for the formation of topological defects.
The topic is timely and the experimental dataset is extensive. The combination of multiple time-resolved probes is potentially powerful and the measurements themselves appear technically sound. However, in its current form the manuscript suffers from issues in presentation, analysis, and interpretation that prevent this referee from recommending publication. In particular, several key claims—especially the identification of topological defects and the separation of amplitude and phase dynamics—are not sufficiently justified by the analysis presented.
The overall structure of the manuscript is difficult to follow. The presentation repeatedly switches between experimental techniques (time-resolved x-ray diffraction, electron diffraction, ARPES, and back to x-ray diffraction) and between measurements at q and q′ without a clear logical progression. As a result, the narrative becomes fragmented and unnecessarily verbose. I strongly encourage the authors to reorganize the manuscript in a clearer way—for example by grouping results according to the physical question addressed (CDW amplitude dynamics, correlation lengths, electronic structure response, etc.) rather than by experimental technique.
Going straight to the scientific discussion, I have several questions that would need to be solved before considering the manuscript for Nat. Comm.
- The manuscript states that the CDW amplitude weakens but does not collapse under a pump fluence of 4 mJ/cm^2. However, inspection of the CDW peaks in Fig. 1a and Fig. 1g suggests markedly different levels of suppression: approximately 20% in one case and nearly 40% in the other. While the authors note that these measurements correspond to different wavevectors (q and q′), such a large discrepancy is unexpected if the measurements probe the same photoexcited volume and if the normalization is consistent. This raises the possibility that the pump and probe volumes differ significantly between measurements, or that the normalization procedure is not equivalent. The authors should clarify this point and discuss whether experimental geometry or penetration depth effects could account for the difference.
- A central claim of the manuscript is that anisotropic peak broadening reflects the formation of topological defects in the CDW. However, anisotropic broadening in reciprocal space simply corresponds to anisotropic correlation lengths in real space. Such behavior can arise from many types of structural disorder, including dislocations, domain walls, strain gradients, or conventional defects. The manuscript does not provide evidence that uniquely identifies the defects as topological. In systems where topological defects play a central role, one typically observes characteristic scaling laws or defect-mediated dynamics (e.g., in the context of KTHNY theory), which are not analyzed here. The authors should either provide stronger evidence supporting the topological interpretation or moderate this claim.
- The discussion regarding the extraction of phase dynamics from x-ray diffraction is difficult to follow and appears conceptually problematic. Since diffraction measures the intensity of the scattered wave (the modulus squared of the structure factor), the phase information of the order parameter is not directly accessible. While phase fluctuations can influence peak widths and correlation lengths, the diffraction signal itself is phase-invariant. It is therefore unclear how the analysis presented allows the authors to distinguish ‘the formation and relaxation timescales of amplitude and phase

degrees of freedom'. This point requires a much clearer explanation and possibly a reformulation of the interpretation. While the experimental dataset is potentially valuable, the central interpretations of the manuscript are not yet convincingly supported. In particular, the identification of topological defects and the separation of amplitude and phase dynamics require stronger justification. I therefore recommend that the authors revise the manuscript and address the points above before it can be considered for publication in Nature Communications.

Reviewer #2

(Remarks to the Author)
The authors aim to study the two co-existing CDW in $EuTe_4$, recently observed, a moiré superstructure formed by two coexisting charge density waves (CDWs) with slightly mismatched wavevectors, resulting in the jointly commensurate CDW (JC-CDW). They used
Different approaches combining tr-XRD, tr-ARPES, and UED offers momentum- and time-resolved monitoring.

1. While the peak position exhibits no significant change upon photoexcitation up to 3.5
125 mJ/cm2 (Figs. 2h): with Electron diffraction the Q-resolution is weak. Could you estimate the Q-resolution obtained in your electron diffraction experiment, known to be not good, especially at high energy?

2. Increase of the transverse width along H: a hallmark of the formation of topological defects suggesting the increasing density of photoinduced topological defects.

I have several comments to make on this topic, which deserves further discussion in the text. First, in temperature-dependent CDW systems, it is common to observe a larger broadening in the transverse direction than in the longitudinal one. In a tight-binding model, which is well-suited to describing RTe_3 systems, the t and t' interactions are strongly anisotropic. In more unidirectional systems ($NbSe_3$, for example), the correlation lengths are always significantly larger in the directions transverse to the chains as one approaches Tc. Compressing the CDW is more costly in terms of Coulomb energy. Your observation of the broadening as a function of H is not surprising.

Second, your claim regarding the creation of transverse topological defects deserves some comments. From a theoretical point of view, a topological object, once created, is stable and resists any form of disorder. However, in your case, the system is reversible (albeit after a long time).
However, the increase in transverse width may also be due to a simple curvature of the wavefronts without affecting the wavelength. This effect has been observed several times in CDW systems affecting mainly the transverse direction [see curvature induced by current: E. Bellec, et al., Phys. Rev. B 101, 125122 (2020) and D. Le Bolloc'het al., Science Advances, 11, 1 (2025)]. This curvature effect is related to the fact that a CDW is strongly pinned to the sample surfaces. I am having difficulty understanding, based on your paper, the position of the surface of your samples and the effect of thickness. If the surfaces do not seem to you to be capable of explaining a curvature of the wavefronts, it could also be a curvature at the edges of CDW grains. An effect involving the creation of CDW domains, followed by coarsening (cited as an explanation in the case of 1T-$TaSe_2$ after a laser pulse: Laulhé et al., Phys. Rev. Lett. 118, 247401 (2017)], combined with a strongly deformed, highly curved CDW within each grain. Shear is not costly since the wavelength remains unchanged.
The topological scenario strikes me as naive. Moreover, the representation in real space of the topological defects you show (FIG 4 h) would imply a tilt in the QCDW vector, which is not the case here.

3. Compared to the rapid nonthermal dynamics of the CDW satellite peaks, the structural Bragg
peaks at (0 0 1) and (0 1 2) exhibit dynamics that are slower by an order of magnitude, which can be attributed to the thermal Debye-Waller effect…

In Fig. S7, the variation in the two Bragg diffraction peaks is far from negligible; it reaches nearly 15% at 4 mJ/cm², whereas the satellite, which certainly has a different dynamic response, barely reaches double that—30%—for the same fluence. This is a very important point. In CDW systems, Bragg reflections are much less sensitive to temperature compared to CDW satellites. How do satellites vary with temperature compared to Bragg reflections in $EuTe_4$? How can we explain that the temperature effect does not affect the CDW given such Bragg behavior ?

Finally, you show that q and the 2q harmonic exhibit similar behavior, with a longer recovery time for 2q. This is somewhat puzzling, since in your introduction you mention a "moiré" pattern consisting of two CDWs that coexist while remaining independent. That does not seem to be the case. Perhaps you could mention the work on $NbSe_3$ in the low temperature phase with the two coexisting (non-parallel) CDWs where a clear echo between the two CDWs is observed under current [A. Ayari et al., Physical Review Letters, 93, 106404 (2004)].
Don't your time-resolved studies ultimately show that you are observing a single object, rather than the coexistence of two distinct waves that interact?

Other comments:

Reference 4 is not complete:
B. Q. et al. Large moiré superstructure of stacked incommensurate charge density waves. Nat. Mater. (2025).
Given that this paper is based primarily on the recent observation of the coexisting CDW in $EuTe_4$ cited in this reference, it is difficult to accept this paper for publication unless the ref 4 has been published.

The structure of the paper is a bit unusual: you treat about UED then XRD, and finally come back to UED again…

Refrence 34, 35 and 36 do not deal with CDW in RTe3 systems but in either YBaCuO or Chromium. Has to be corrected.

Fig.3D : Is there a color error in the legend?

Fig. 3B : The scheme must be changed because this is transverse fluctuations

Fig. 3E : is it a fit ? From which data ?

In conclusion, I personally find the research topic interesting, and I am impressed by the number of experimental techniques used. However, I find the results—though well executed—somewhat disappointing in terms of their physical significance. The interpretation could also be further developed.

Reviewer #3

(Remarks to the Author)
In this work, Oh, Su, Ning and coworkers investigate the ultrafast dynamics of the jointly-commensurate charge density wave (JC-CDW) formed in EuTe4 by using static hard X-ray diffraction in conjunction with time-resolved soft X-ray/ electron diffraction and ARPES. This paper builds on an earlier work from the group that demonstrated the existence of the JC-CDW which describes a moiré-like charge modulation, due to the slight wavevector mismatch between the incommensurate CDWs comprising it (Ref. 4 of the main text).
The present paper adds important insight into the non-equilibrium behavior of the accompanying lattice distortion. The key observation is an unusual anisotropic spot broadening, perpendicular to the monolayer CDW wavevector. The authors assign it to a shear-type topological defect and conclude it to be a characteristic feature of the JC-CDW that arises from the wavevector-locking to the underlying lattice, reminiscent of commensurate CDWs. They further extend the discussion to photoinduced CDW amplitude and phase modulations and develop an analysis of first- and second-order satellite spot intensities in tr-XRD to disentangle their dynamics. The approach is validated by cross-checking with tr-ARPES and UED signatures which are directly sensitive to amplitude and phase fluctuations, respectively.
I am convinced by the validity of the results and the balanced conclusions that are drawn from the data. The observation of shear-type topological defect, directly tied to the formation of the JC-CDW is new and of general interest to the broad audience of Nature Communications. While questions regarding the exact formation mechanism of the shear-type defects remain, I generally support publication after the authors have addressed the below comments:
(1) Optical moiré engineering: The authors motivate their study specifically by the possibility to optically tune moiré potentials by harnessing the susceptibility of charge-ordered states towards photoexcitation. The wording in abstract and conclusions give the reader the impression that optical tunability is a unique property of JC-CDWs and there is no mention of the photoinduced modulation in twist angle that was shown by Duncan et. al. (Nature 647, 619–624 (2025)). The statements in the text should reflect the state of research in this regard.
(2) Figure 1: In the caption it is mentioned that “I denotes the scattering intensity”, yet there is no intensity scale in the figure.
(3) Figure 1: The figure shows an artistic representation of the material structure but the formation of the JC-CDW from I-CDWs in different tellurium planes is not directly obvious. A visual representation of this somewhere in the manuscript would make the concept more accessible to the reader without having to look up Ref. 4.
(4) Polarization dependence: As the employed excitation energies in UED and ARPES measurements lie close to the CDW gaps of the material, indicated in Fig. S8, one could expect strongly anisotropic optical properties with respect to the periodic lattice distortion. Similarly, electron-phonon coupling may depend on the excited electronic states. As the polarization is only specified in the XRD methods section, I would like the authors to comment, if they observed a polarization dependence and if they used the same polarization throughout all experiments.
(5) Bilayer CDW detection in UED: In line 122, it is stated that the bilayer CDW is not detectable in UED experiments. This point is not further commented and thus not directly obvious to a non-expert reader.

Version 1:

Reviewer comments:

Reviewer #1

(Remarks to the Author)
The authors have provided a detailed response to my queries. Besides, refurbishing the paper, which now reads smoothly, they give a comprehensive response to my concerns. Indeed, some of my questions were also raised the the other´s referees. I, therefore, recommend the publication of the manuscript in Nat. Comm.

Reviewer #2

(Remarks to the Author)

Open Access This Peer Review File is licensed under a Creative Commons Attribution 4.0 International License, which permits use, sharing, adaptation, distribution and reproduction in any medium or format, as long as you give appropriate credit to the original author(s) and the source, provide a link to the Creative Commons license, and indicate if changes were made.
In cases where reviewers are anonymous, credit should be given to 'Anonymous Referee' and the source.
The images or other third party material in this Peer Review File are included in the article's Creative Commons license, unless indicated otherwise in a credit line to the material. If material is not included in the article's Creative Commons license and your intended use is not permitted by statutory regulation or exceeds the permitted use, you will need to obtain permission directly from the copyright holder.
To view a copy of this license, visit https://creativecommons.org/licenses/by/4.0/

Reviewer #1 (Remarks to the Author):

This manuscript reports time-resolved studies of the charge density wave (CDW) dynamics in $EuTe_4$ using a combination of ultrafast x-ray diffraction, electron diffraction, and time-resolved ARPES. The CDW in this material forms a moiré superstructure resulting from two incommensurate CDWs. The authors report that photoexcitation reduces the CDW amplitude while the diffraction peaks develop an anisotropic broadening, which they interpret as evidence for the formation of topological defects.

The topic is timely and the experimental dataset is extensive. The combination of multiple time-resolved probes is potentially powerful and the measurements themselves appear technically sound.

However, in its current form the manuscript suffers from issues in presentation, analysis, and interpretation that prevent this referee from recommending publication. In particular, several key claims—especially the identification of topological defects and the separation of amplitude and phase dynamics—are not sufficiently justified by the analysis presented.

Our reply:
We thank the Reviewer for providing a precise summary, positive assessment, and critical feedback on our manuscript. We agree that the presentation, analysis, and interpretation in the previous version of the manuscript can be improved. Following a major revision, we believe that these concerns have now been fully addressed, as discussed in detail below.

The overall structure of the manuscript is difficult to follow. The presentation repeatedly switches between experimental techniques (time-resolved x-ray diffraction, electron diffraction, ARPES, and back to x-ray diffraction) and between measurements at q and q′ without a clear logical progression. As a result, the narrative becomes fragmented and unnecessarily verbose. I strongly encourage the authors to reorganize the manuscript in a clearer way—for example by grouping results according to the physical question addressed (CDW amplitude dynamics, correlation lengths, electronic structure response, etc.) rather than by experimental technique.

Our reply:
We thank the Reviewer for suggesting ways to improve the organization and structure of our manuscript. As suggested by the Reviewer, **we have significantly revised the presentation by grouping the results based on the physical questions addressed** : (1) Static momentum space mapping, (2) CDW amplitude and phase fluctuation dynamics, and (3) CDW correlation length and periodicity dynamics. This reorganization reduces the frequent switching between different

experimental techniques and provides a clearer logical progression through the findings in the order of tr-XRD → tr-ARPES → UED. Moreover, **we have substantially improved Figs. 1–3 with sufficient schematic illustrations in the main text to facilitate a clearer understanding of the content**. We believe that the restructured version of the manuscript is now more succinct, coherent, and logically organized.

Going straight to the scientific discussion, I have several questions that would need to be solved before considering the manuscript for Nat. Comm.

- The manuscript states that the CDW amplitude weakens but does not collapse under a pump fluence of 4 mJ/cm². However, inspection of the CDW peaks in Fig. 1a and Fig. 1g suggests markedly different levels of suppression: approximately 20% in one case and nearly 40% in the other. While the authors note that these measurements correspond to different wavevectors (q and q′), such a large discrepancy is unexpected if the measurements probe the same photoexcited volume and if the normalization is consistent. This raises the possibility that the pump and probe volumes differ significantly between measurements, or that the normalization procedure is not equivalent. The authors should clarify this point and discuss whether experimental geometry or penetration depth effects could account for the difference.

Our reply:

The Reviewer has correctly pointed out the different levels of suppression between Fig. 2a and Fig. 2g. We would like to first clarify that both measurements correspond to the same monolayer CDW wavevector q, rather than to q' (denoted as q_1 and q_2 in the revised manuscript). This mismatch in suppression level arises from a sizable background that was not subtracted in the previous version of **Figs. 2a and 2d**. This is because the purpose of these figures is to demonstrate the absence of peak shifts and broadening, rather than to quantify the magnitude of the intensity suppression. **We have now revised Figs. 2a and 2d (new Figs. 3a and 3d) by subtracting the uniform background from the diffraction peaks, as reproduced in Fig. R1.** After background subtraction and normalization of the unpumped diffraction intensity to unity, both measurements show a consistent intensity drop of ~35%. This suppression level is also consistent with time traces measured by tr-XRD (new Fig. 2c) and UED (new Fig. S8a), where the background has been subtracted. Moreover, the uniform background of the UED peak profiles has also been properly subtracted (new Fig. 3g).

We have also considered whether pump-probe volume mismatch could contribute to our measurements. Laterally, we have ensured that the pump spot size is consistently ~4 times larger than the probe spot size for all diffraction measurements and diffraction peaks, minimizing any pump-probe overlap mismatch. Regarding the penetration depth mismatch, the consistent suppression across UED and tr-XRD measurements suggests that such an effect is insignificant in

our measurements. This is because our UED experiment was performed on ~50 nm-thick samples in a transmission geometry. Since such a small thickness is comparable to, if not smaller than, the pump optical penetration depth, there is no mismatch issue in the UED experiment. The similar suppression level observed in tr-XRD thus supports the conclusion that penetration-depth mismatch is also negligible in the tr-XRD experiment. **In the revised manuscript, we have expanded Supplementary Note 2 (new Supplementary Note 5) to discuss the pump-probe volume overlap and penetration-depth effects in detail.**

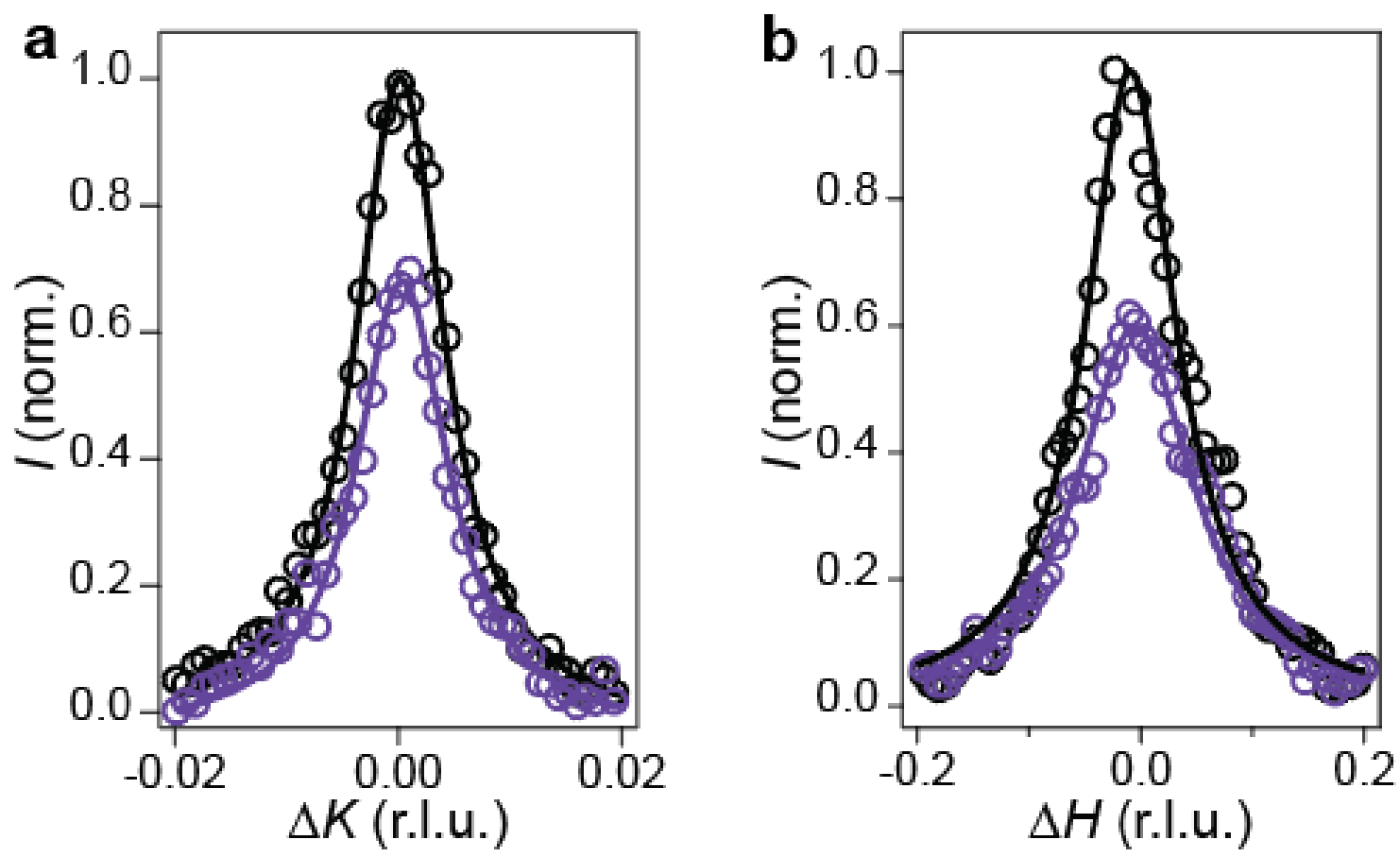

Fig. R1. **a**, *K*-cut of the $\mathbf{q}_1$ peak at (0 -0.644 1), taken before photoexcitation (black) and at the time delay corresponding to the maximum suppression of the peak intensity (purple), with $F = 4$ mJ/cm^2 from tr-XRD. **b**, *H*-cut of the $\mathbf{q}_1$ peak at (3 0.356 0), taken before photoexcitation (black) and at the time delay corresponding to the maximum suppression of the peak intensity (purple), with $F = 4$ mJ/cm^2 from UED.

- A central claim of the manuscript is that anisotropic peak broadening reflects the formation of topological defects in the CDW. However, anisotropic broadening in reciprocal space simply corresponds to anisotropic correlation lengths in real space. Such behavior can arise from many types of structural disorder, including dislocations, domain walls, strain gradients, or conventional defects.

Our reply:

As the Reviewer pointed out, we attribute the transverse broadening of the CDW diffraction peak to shear-type CDW defects rather than to structural disorder or conventional crystalline defects. This assignment is based on the observation that the broadening of the CDW diffraction peaks along the *H* direction (new Fig. S8i) is ~10 times larger than that of the structural Bragg peaks measured along the same direction (new Fig. S3e), as reproduced in Fig. R2. The comparatively

weak broadening of the structural Bragg peaks can be attributed to conventional structural disorder and defects, whereas the much larger broadening of the CDW peak indicates dominant disorder associated specifically with the CDW order. **We have included a concise version of this discussion in the main text and a more detailed discussion in a new Supplementary Note 9.**

We further note that anisotropic broadening of the CDW diffraction peak is not naturally expected in quasi-2D telluride CDWs with only weak structural orthorhombicity, as evidenced by the isotropic broadening of the CDW peaks along both in-plane directions in the structurally similar rare-earth tritellurides $R\mathrm{Te}_3$ (Moore et al., *Phys. Rev. B* 93, 024304 (2016); Zong et al., *Nat. Phys.* 15, 27–31 (2019)). This point is discussed in detail in our response to Referee 2's comments.

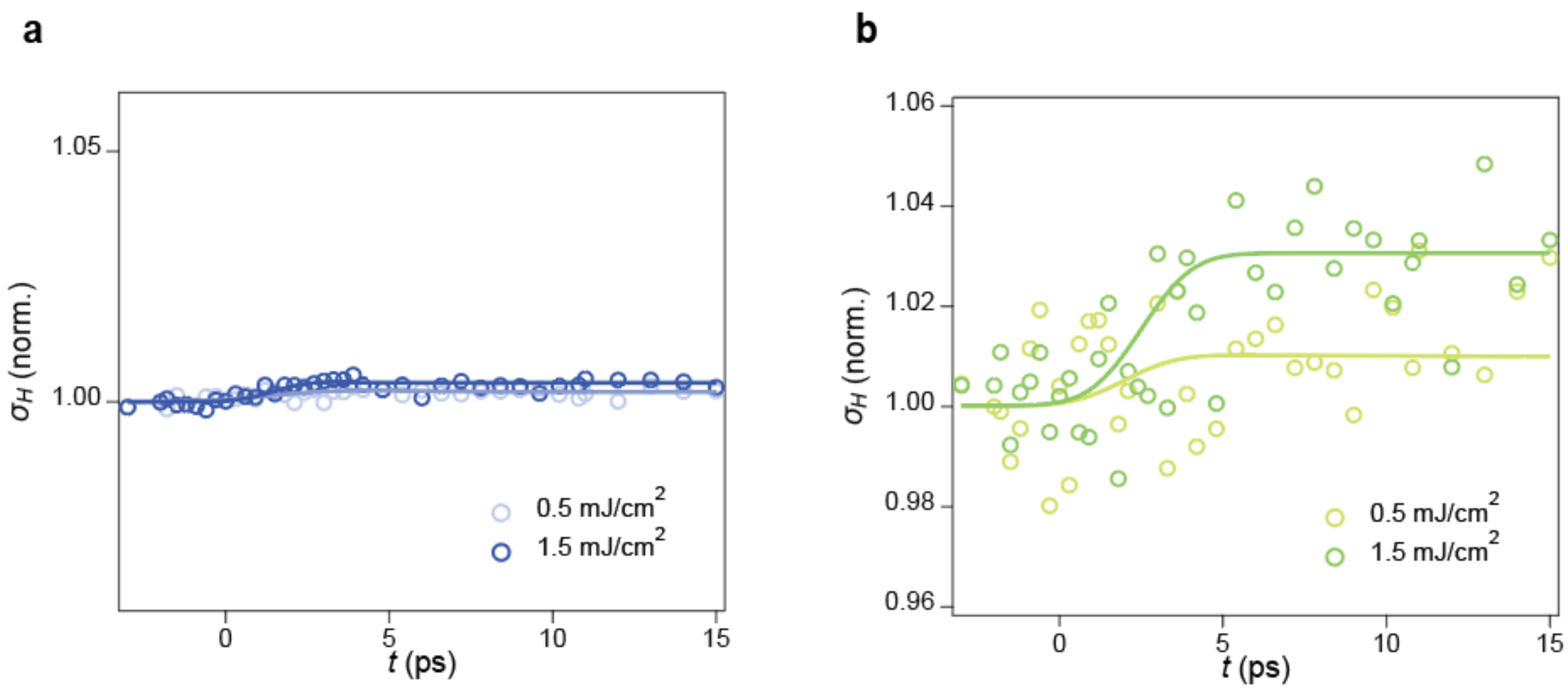

Fig. R2. Temporal evolution of the **a**, structural Bragg peak width and **b**, CDW satellite peak width, averaged over multiple peaks in the UED measurements. σ_H is the statistical standard deviation of the diffraction peak profile along the *H* direction. All data are normalized to their equilibrium values and fitted with a single exponential decay plus a constant offset as shown by the solid lines. Figures **a, b** are reproduced from the new Fig. S3e and Fig. S8i, respectively.

The manuscript does not provide evidence that uniquely identifies the defects as topological. In systems where topological defects play a central role, one typically observes characteristic scaling laws or defect-mediated dynamics (e.g., in the context of KTHNY theory), which are not analyzed here. The authors should either provide stronger evidence supporting the topological interpretation or moderate this claim.

Our reply:

We agree with the Reviewer that more rigorous analyses, such as identifying characteristic scaling laws or defect-mediated dynamics consistent with KTHNY theory, would be valuable for definitively establishing their topological character. However, we kindly note that the formation of topological defects in photoexcited CDW systems is already a widely accepted concept in the literature, rather than a specific claim introduced in our work. Indirect evidence purely from ultrafast CDW dynamics dates back to transient optical reflectivity experiments (Yusupov et al., *Nat. Phys.* 6, 681 (2010); Mertelj et al., *Phys. Rev. Lett.* 110, 156401 (2013)). More recently, a number of static and time-resolved diffraction studies similar to our work, without performing rigorous analyses based on scaling laws or KTHNY theory, have explicitly attributed the broadening of CDW diffraction peaks to the formation of topological defects (Vogelgesang et al., *Nat. Phys.* 14, 184 (2018); Zong et al., *Nat. Phys.* 15, 27 (2019); Zhou et al., *Nat. Commun.* 12, 1, 566 (2021); Cheng et al., *Nat. Phys.* 20, 54 (2024)).

That said, we agree with the Reviewer that the topological nature of the light-induced CDW defects should be more rigorously verified, which is beyond the scope of the present work. Since whether the light-induced shear defects are truly topological is not a central claim of this manuscript, **we have removed the term "topological" when describing the defects inferred from our experimental data as suggested by the Reviewer.**

- The discussion regarding the extraction of phase dynamics from x-ray diffraction is difficult to follow and appears conceptually problematic. Since diffraction measures the intensity of the scattered wave (the modulus squared of the structure factor), the phase information of the order parameter is not directly accessible. While phase fluctuations can influence peak widths and correlation lengths, the diffraction signal itself is phase-invariant. It is therefore unclear how the analysis presented allows the authors to distinguish 'the formation and relaxation timescales of amplitude and phase degrees of freedom'. This point requires a much clearer explanation and possibly a reformulation of the interpretation.

Our reply:

The Reviewer is correct that obtaining the phase dynamics from diffraction intensity is not straightforward. While the absolute phase of the order parameter is not directly observable in conventional diffraction, the diffraction intensity is sensitive to the *phase fluctuations* of the CDW order parameter. This framework was derived in several pioneering works on diffraction of CDWs (Overhauser, *Phys. Rev. B* 3, 3173 (1971); Lee et al., *Nat. Commun.* 3, 838 (2012)). In analogy to the Debye-Waller effect, where incoherent phonons suppress the coherent peak intensity, the CDW phase fluctuations ($\Delta\varphi^2$) suppress the diffraction intensity by the factor of $\exp(-A*\Delta\varphi^2)$, where A is a constant. In our manuscript, we also explicitly presented the diffraction intensity of first-order and second-order CDW satellite peaks as functions of both CDW amplitude and phase fluctuations.

$$I_1 \sim A^2 e^{-\frac{\Delta\phi^2}{2}} \tag{1}$$

$$I_2 \sim (A^2 + \gamma A)^2 e^{-2\Delta\phi^2} \tag{2}$$

In order to clarify the workflow through which we extract phase information from peak intensity, **we have made several improvements in the manuscript to address this subtlety**:

1. **We have revised Fig. 1 (as reproduced in Fig. R3) and added a paragraph** to illustrate the correspondence between the CDW physical quantities studied and techniques employed;
2. **We have explicitly drawn an analogy to the Debye-Waller effect and made the formula to extract the CDW amplitude and phase fluctuation dynamics from diffraction intensities I_1 and I_2 as stand-alone equations** as shown in Eqs. (1),(2).
3. Following the formula mentioned above, **we have added a schematic of the workflow for extracting amplitude and phase fluctuations from the intensity of multiple peaks in the new Fig. 2a,b (as reproduced in Fig. R4):**

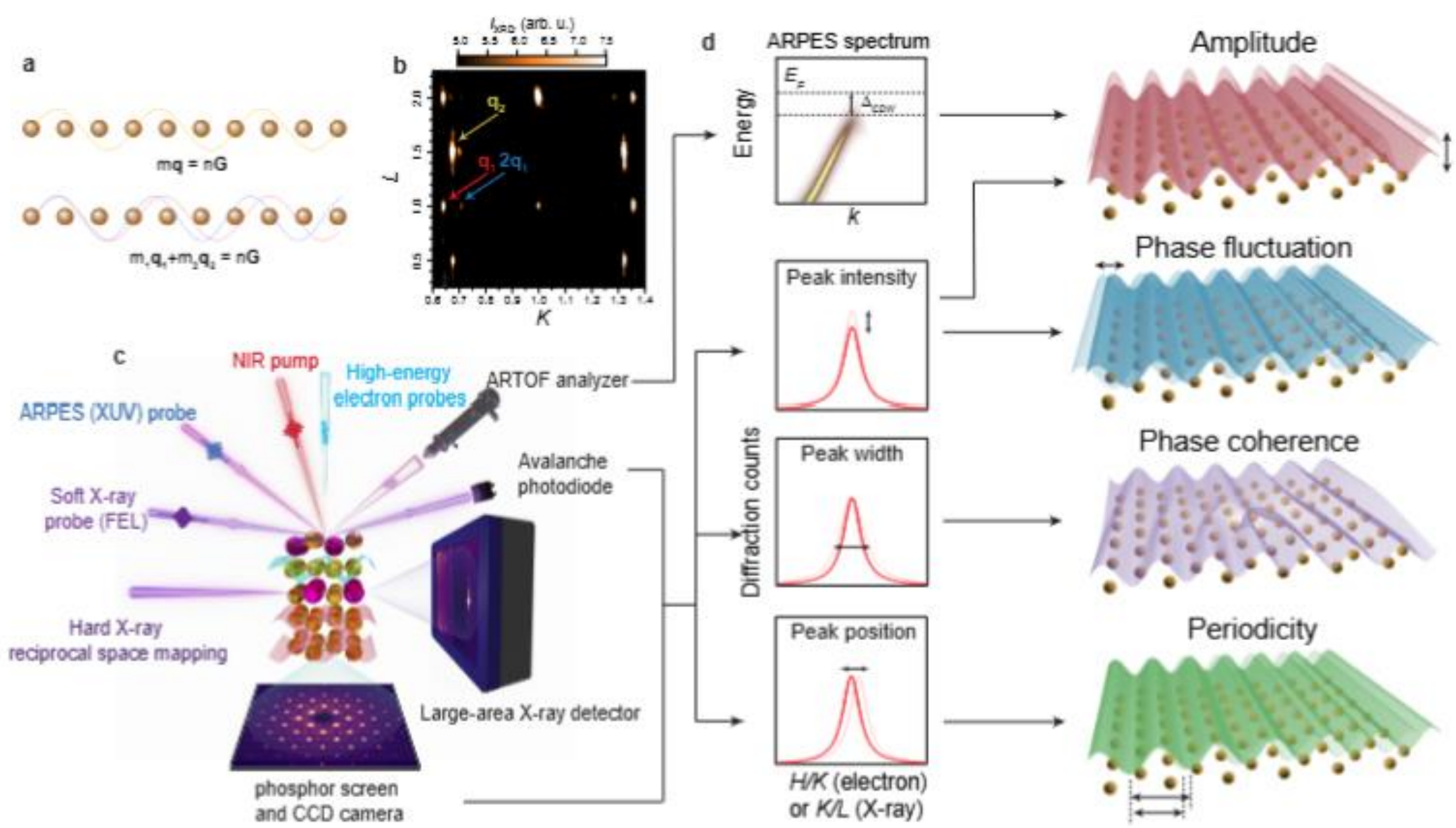

Fig. R3. Correspondence between the investigated CDW physical quantities and the experimental techniques employed.

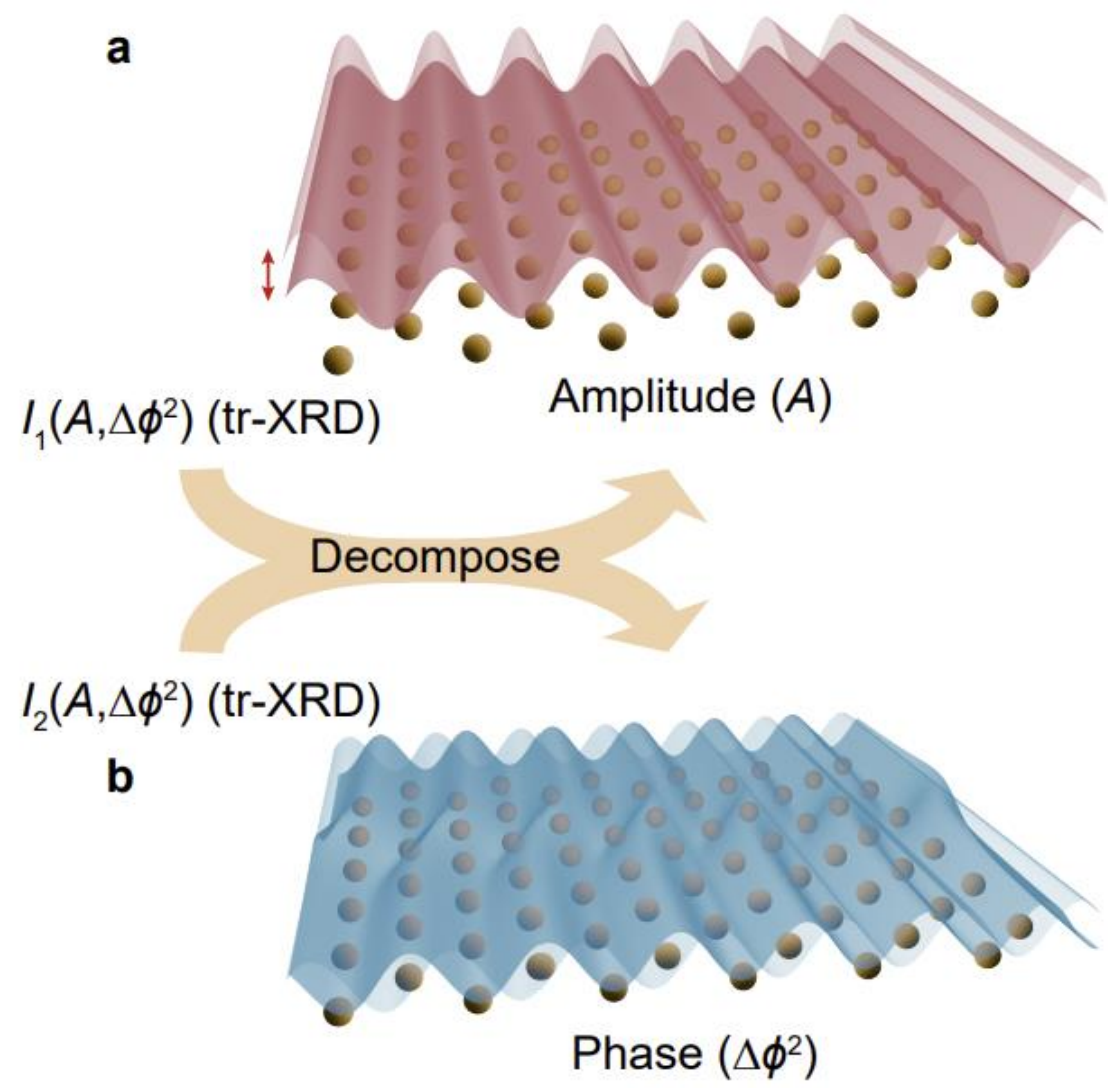

Fig. R4. Schematics of the workflow for extracting amplitude and phase fluctuation dynamics.

While the experimental dataset is potentially valuable, the central interpretations of the manuscript are not yet convincingly supported. In particular, the identification of topological defects and the separation of amplitude and phase dynamics require stronger justification. I therefore recommend that the authors revise the manuscript and address the points above before it can be considered for publication in Nature Communications.

Our reply:

We thank the Reviewer for recognizing the potential value of the experimental dataset. We believe that the concerns raised by the Referee have now been fully addressed.

Reviewer #2 (Remarks to the Author):

The authors aim to study the two co-existing CDW in EuTe4, recently observed, a moiré superstructure formed by two coexisting charge density waves (CDWs) with slightly mismatched wavevectors, resulting in the jointly commensurate CDW (JC-CDW). They used different approaches combining tr-XRD, tr-ARPES, and UED offers momentum- and time-resolved monitoring.

1. While the peak position exhibits no significant change upon photoexcitation up to 3.5125 mJ/cm2 (Figs. 2h): with Electron diffraction the Q-resolution is weak. Could you estimate the Q-resolution obtained in your electron diffraction experiment, known to be not good, especially at high energy?

Our reply:
The nominal resolution of our 26 keV UED system, given by the full width half maximum (FWHM) of the raw electron beam, is approximately 0.03 $Å^{-1}$, which corresponds to 0.02 in-plane reciprocal lattice unit in $EuTe_4$. However, a typical Lorentzian fitting could easily return super-resolution information down to ~10^{-5} $Å^{-1}$ as we demonstrated before using the same setup (*Nano Lett.* 23, 10772–10778 (2023)). We thus believe the resolution of our keV UED systems is good enough to support the claim in the present work. **We have now added the discussion of q-resolution of the keV UED setup in the Methods section.**

2. Increase of the transverse width along H: a hallmark of the formation of topological defects suggesting the increasing density of photoinduced topological defects.

I have several comments to make on this topic, which deserves further discussion in the text. First, in temperature-dependent CDW systems, it is common to observe a larger broadening in the transverse direction than in the longitudinal one. In a tight-binding model, which is well-suited to describing RTe3 systems, the t and t' interactions are strongly anisotropic. In more unidirectional systems (NbSe3, for example), the correlation lengths are always significantly larger in the directions transverse to the chains as one approaches Tc. Compressing the CDW is more costly in terms of Coulomb energy. Your observation of the broadening as a function of H is not surprising.

Our reply:
The Reviewer raises an important point regarding whether transverse broadening of CDW peaks in $EuTe_4$ should be considered unexpected given its structural and electronic in-plane anisotropy. We have clarified in the main text that why the transient anisotropic broadening is **not** an obvious

consequence of electronic hopping anisotropy and can**not** be readily inferred from equilibrium anisotropy.

First, the anisotropy of a CDW diffraction peak is governed by the CDW phase stiffness, or equivalently by the CDW correlation length, and cannot be directly inferred from the anisotropy of the single-particle hopping parameters. Although the hopping parameters t and t', which correspond to directions parallel and perpendicular to the in-plane Te 5p orbitals, respectively, differ substantially by up to a factor of 5 in $R\mathrm{Te}_3$ (Rettig et al., *Nat. Commun.* 7, 10459 (2016)), this does not necessarily imply a comparably strong anisotropy of CDW diffraction peaks. In fact, high-resolution XRD, ED, and STM studies on CeTe_3, ErTe_3, and EuTe_4 have reported no clear signatures of strong anisotropy in the CDW diffraction peaks (Okuma et al., arXiv:2604.25436, Mallayya et al., *Nat. Phys.* 20, 822–829 (2024); Wu et al., *Phys. Rev. Mater.* 3, 024002 (2019), Xiao et al., *Nano Lett.* 24, 7681–7687 (2024)). This indicates that strong hopping anisotropy does not, by itself, imply strongly anisotropic CDW peak broadening in these quasi-2D tellurides.

This is also why the comparison with NbSe_3 is not straightforward. NbSe_3 has a quasi-1D lattice structure, where the phase stiffness is intrinsically very different along and transverse to the chain direction, as reflected in the pronounced anisotropy of the correlation length and CDW diffraction peak widths, which can be more than tenfold (Prodan et al., *Solid State Commun.* 150, 2134 (2010), Pinsolle et al., *Phys. Rev. Lett.* 109, 256402 (2012)). By contrast, EuTe_4 and related $R\mathrm{Te}_3$ are quasi-2D with relatively small structural orthorhombicity and modest anisotropy in correlation lengths. Thus, the transverse broadening observed in EuTe_4 should not be regarded as an obvious consequence of the same physics that governs quasi-1D CDWs.

Second, the presence of equilibrium anisotropy also does not necessarily imply nonequilibrium anisotropy. This point is supported by the comparison between EuTe_4 and $R\mathrm{Te}_3$. While they share similar lattice structures, anisotropic hopping parameters, and nearly isotropic equilibrium CDW peak widths, their photoinduced CDW coherence dynamics are qualitatively different. $R\mathrm{Te}_3$, which hosts a single incommensurate CDW, exhibits isotropic broadening of the CDW peaks along both in-plane directions (Moore et al., *Phys. Rev. B* 93, 024304 (2016); Zong et al., *Nat. Phys.* 15, 27–31 (2019)). By contrast, EuTe_4, which hosts two jointly commensurate incommensurate CDWs, shows selective broadening of the CDW peaks primarily along the direction perpendicular to the CDW wavevector. This qualitative difference suggests that the broadening in EuTe_4 is not a generic consequence of the underlying structural or electronic anisotropy, but is instead tied to the joint commensuration of the two incommensurate CDWs.

We further note that this broadening is unlikely to originate from simple lattice heating toward the CDW transition temperature. In both EuTe_4 and LaTe_3, the CDW transition temperature exceeds 600–700 K, while the pump-induced lattice heating under the maximum fluence used in this study is estimated to be only on the order of several tens of kelvin (Zong et al., *Nat. Phys.* 15, 27–31 (2019)). The sample therefore remains far below the transition temperature, making conventional thermal critical broadening unlikely.

Taken together, these considerations indicate that the anisotropic broadening of CDW peaks observed in $EuTe_4$ is not expected from hopping anisotropy, structural anisotropy, or simple heating alone. Rather, it reflects a nonequilibrium reduction of the transverse CDW phase correlation length associated with the joint commensuration. **We have included a concise version of this discussion in the main text and a more detailed discussion in a new Supplementary Note 9.**

Second, your claim regarding the creation of transverse topological defects deserves some comments. From a theoretical point of view, a topological object, once created, is stable and resists any form of disorder. However, in your case, the system is reversible (albeit after a long time).

Our reply:
We agree that topological objects should be robust once created. However, a number of previous studies have reported the annihilation of light-induced topological defects in CDWs faster than nanosecond timescales, such that full recovery to the pre-pumped CDW state occurs before the arrival of the next optical pulse (Lee et al., *Nat. Commun.* 3, 838 (2012); Vogelgesang et al., *Nat. Phys.* 14, 184 (2018); Zong et al., *Nat. Phys.* 15, 27 (2019); Zhou et al., *Nat. Commun.* 12, 1, 566 (2021); Cheng et al., *Nat. Phys.* 20, 54 (2024)). That said, we recognize that demonstrating the topological nature of the light-induced CDW defects requires rigorous imaging or scaling law analyses, which are beyond the scope of this work. **To this end, we have removed the term "topological" when describing the defects inferred from our experimental data as suggested by the Reviewer.**

However, the increase in transverse width may also be due to a simple curvature of the wavefronts without affecting the wavelength. This effect has been observed several times in CDW systems affecting mainly the transverse direction [see curvature induced by current: E. Bellec, et al., Phys. Rev. B 101, 125122 (2020) and D. Le Bolloc'het al., Science Advances, 11, 1 (2025)]. This curvature effect is related to the fact that a CDW is strongly pinned to the sample surfaces. I am having difficulty understanding, based on your paper, the position of the surface of your samples and the effect of thickness. If the surfaces do not seem to you to be capable of explaining a curvature of the wavefronts, it could also be a curvature at the edges of CDW grains. An effect involving the creation of CDW domains, followed by coarsening (cited as an explanation in the case of 1T-$TaSe_2$ after a laser pulse: Laulhé et al., Phys. Rev. Lett. 118, 247401 (2017)], combined with a strongly deformed, highly curved CDW within each grain. Shear is not costly since the wavelength remains unchanged.

Our reply:

The Reviewer raises an important point regarding whether the selective transverse broadening could arise from CDW wavefront curvature rather than from shear-type defects. We agree that a curved CDW wavefront would primarily broaden the CDW peak in the transverse direction without necessarily changing the CDW wavelength, and we have added a discussion of this possibility in the revised manuscript.

We first note that the mechanism reported for current-induced CDW bending in $NbSe_3$ is unlikely to apply directly here. In the cited studies, wavefront bending occurs in a quasi-1D CDW system under a unidirectional DC current applied along the CDW wavevector. By contrast, $EuTe_4$ is a quasi-2D system with only weak in-plane structural anisotropy, and our experiment uses above-bandgap optical excitation rather than a DC current. Unlike a directional sliding force, the optical pump generates nonequilibrium carriers followed by incoherent thermalization that largely erases the initial pump polarization information. Therefore, the two key conditions underlying the CDW bending—strong intrinsic lattice anisotropy and a unidirectional bias—are not fulfilled in our experiment. Consistently, a previous study by the same authors (Le Bolloc'h et al., *Phys. Rev. B* 93, 165124 (2016)) found that the quasi-2D $TbTe_3$ does not exhibit clear anisotropic peak broadening under similar DC-current-driven conditions (Fig. R5). Since $EuTe_4$ is structurally much closer to $TbTe_3$ than to $NbSe_3$, the $NbSe_3$-type current-induced macroscopic bending mechanism is unlikely to be the dominant origin of the broadening observed here.

[Redacted]

Fig. R5. Coherent diffraction patterns of the CDW satellite reflection as a function of external current, below and above the threshold current I_S, where the CDW structure undergoes a drastic deformation, in (a) $TbTe_3$ and (b) $NbSe_3$. Adapted from *Phys. Rev. B* 93, 165124 (2016).

We next consider whether surface or edge pinning could produce a macroscopic curvature of the CDW wavefront. This scenario is strongly constrained by the experimentally observed timescale. The CDW peak width reaches its maximum within approximately 3 ps. Taking a typical sound velocity of ~2500 m/s in CDW tellurides, a smooth CDW deformation can propagate only over ~7.5 nm within this time. Therefore, it is unlikely that a curvature profile could develop across an entire CDW domain (domain size ~100 nm) on this time scale.

A more local version of the Reviewer's proposed scenario, namely bending near pre-existing CDW grain/domain boundaries, remains possible. However, in this local limit, such curvature is phenomenologically equivalent to a shear-type CDW defect: it locally distorts the CDW phase in the transverse direction while leaving the CDW wavelength unchanged. Since our probe beam spot size (~100 um) is much larger than the domain size (~100 nm), we cannot determine whether these shear distortions nucleate specifically at pre-existing CDW domain boundaries or at more randomly distributed positions within the pumped region. Nevertheless, both scenarios support the same conclusion that photoexcitation produces a shear-like disruption of the transverse CDW correlation length.

Importantly, even if the resulting shear-like deformation is phenomenologically similar to the CDW wavefront bending reported under DC-current-driven conditions, our observation establishes that such shear-type CDW distortions can be generated on ultrafast timescales by optical excitation, which, to the best of our knowledge, has not been reported previously. This distinguishes our work from earlier current-driven studies and highlights a nonequilibrium route for generating shear-like CDW defects through femtosecond photoexcitation.

Therefore, we believe that the formation of inhomogeneous CDW defects, as suggested in a number of photoexcited CDW studies (Laulhé et al., *Phys. Rev. Lett.* 118, 247401 (2017); Cheng et al., *Nat. Phys.* 20, 54–60 (2024); Zong et al., *Nat. Phys.* 15, 27–31 (2019); Zhou et al., *Nat. Commun.* 12, 566 (2021)), is consistent with our observations. **We have revised the main text and included a new Supplementary Note 9 to clarify that macroscopic surface- or edge-pinning-induced wavefront curvature is unlikely to explain the observed ultrafast broadening, whereas local curvature near CDW domain boundaries is compatible with our interpretation of photoinduced shear-type CDW defects.**

The topological scenario strikes me as naive. Moreover, the representation in real space of the topological defects you show (FIG 4 h) would imply a tilt in the QCDW vector, which is not the case here.

Our reply:

We respectfully point out that the emergence of shear-type CDW defects would not result in a tilt of $\mathbf{q}_{CDW}$, in agreement with our observations. First, left- and right-bent configurations are expected to occur with equal probability on a macroscopic scale, as there is no inherent preference over one. Consequently, the equal population of these two types of defects averages out and does not produce a measurable tilt of $\mathbf{q}_{CDW}$. Second, even if the populations are not exactly equal, we have shown above that the shear-type CDW defects or local bending remain highly localized on few-nm length scales, which would not produce a clearly tilted $\mathbf{q}_{CDW}$ given a micron scale probe beam spot size. Instead, they would primarily manifest as a selective loss of CDW coherence perpendicular to $\mathbf{q}_{CDW}$, consistent with the observed transverse peak broadening.

In the previous Fig. 4, only one type of shear defects was illustrated for visual clarity. **We have recognized that this schematic could be misleading and revised Fig. 4 to explicitly include both types of defects that can be generated upon photoexcitation**.

3. Compared to the rapid nonthermal dynamics of the CDW satellite peaks, the structural Bragg

peaks at (0 0 1) and (0 1 2) exhibit dynamics that are slower by an order of magnitude, which can be attributed to the thermal Debye-Waller effect…

In Fig. S7, the variation in the two Bragg diffraction peaks is far from negligible; it reaches nearly 15% at 4 mJ/cm², whereas the satellite, which certainly has a different dynamic response, barely reaches double that—30%—for the same fluence. This is a very important point. In CDW systems, Bragg reflections are much less sensitive to temperature compared to CDW satellites. How do satellites vary with temperature compared to Bragg reflections in EuTe4 ? How can we explain that the temperature effect does not affect the CDW given such Bragg behavior?

Our reply:

We agree with the Reviewer that CDW peaks are generally expected to be more sensitive to pulsed laser excitation than lattice Bragg peaks. However, their quantitative difference varies vastly from material to material and from peak to peak. To ensure a direct comparison and understand whether the Debye-Waller effect is manifested in both the CDW and Bragg dynamics, we average several Bragg peaks and their neighboring CDW peaks and compare their responses under the same pump fluences of 0.5 and 1.5 mJ/cm^2 measured by UED. We choose fluences below 1.5 mJ/cm^2 because the CDW dynamics saturate above this critical fluence (new Fig. S8a and *Phys. Rev. Lett.* 135, 246504 (2025)) and thus study the thermal effect in the linear regime.

As shown in Fig. R6, at long time delays > 20 ps, the intensity suppression of both the Bragg and CDW peaks is affected by the Debye-Waller effect. In this regime, the relative intensity drop of the Bragg peak is approximately 50% of that for the CDW peak. This is qualitatively consistent

with their static temperature dependence (Fig. R7), where the relative decrease in the Bragg peak intensity is also about half of that of the CDW peak near 300 K on the heating branch. We further note that the relatively modest CDW peak suppression may reflect the exceptional robustness of the joint commensuration against photoexcitation.

In summary, the "temperature effect" manifests itself both in CDW and Bragg dynamics in long timescales, while the short-timescale dynamics of the CDW peak, the focus of the manuscript, is nonthermal, and such an effect is consistent with their static temperature dependence. **We have now included this discussion in Supplementary Note 8 (new Supplementary Note 3) .**

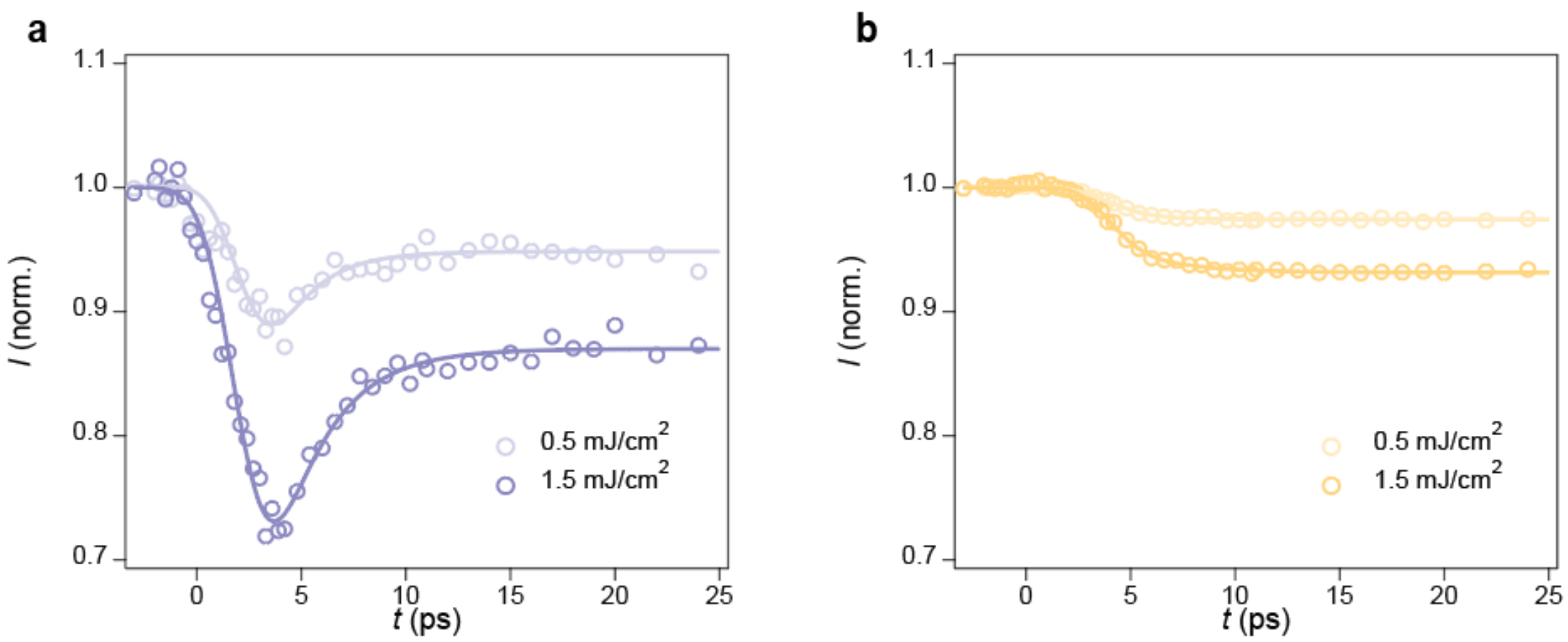

Fig. R6. Temporal evolution of the **a** CDW and **b** Bragg peak intensities at pump fluences of 0.5 and 1.5 mJ/cm^2 measured by UED. Figures **a, b** are reproduced from the new Fig. S8a and Fig. S3c, respectively.

[Redacted]

Fig. R7. Temperature dependence of CDW satellite peak (0, -1.644, 10) (left) and lattice Bragg peak (0, -2, 10) (Right) measured in synchrotron-based X-ray diffraction. Figures are adapted from *Phys. Rev. Lett.* 128, 036401 (2024).

Finally, you show that q and the 2q harmonic exhibit similar behavior, with a longer recovery time for 2q. This is somewhat puzzling, since in your introduction you mention a “moiré” pattern consisting of two CDWs that coexist while remaining independent. That does not seem to be the case. Perhaps you could mention the work on NbSe3 in the low temperature phase with the two coexisting (non-parallel) CDWs where a clear echo between the two CDWs is observed under current [A. Ayari et al., Physical Review Letters, 93, 106404 (2004)].

Don’t your time-resolved studies ultimately show that you are observing a single object, rather than the coexistence of two distinct waves that interact?

Our reply:
We apologize for the confusion regarding the labelling of different CDW orders and peaks. We clarify here that the moiré superlattice is formed between the monolayer CDW **q** and the bilayer CDW **q’**, instead of between the **q** and 2**q** peaks, which denote the first- and second-order diffraction peaks of the same monolayer CDW.

To avoid further confusion, we have relabelled these three quantities as follows across the revised manuscript:

- The first-order diffraction of the monolayer CDW : $\mathbf{q}_1$ (previously **q**)
- The second-order diffraction of the monolayer CDW : $2\mathbf{q}_1$ (previously 2**q**)
- The first-order diffraction of the bilayer CDW : $\mathbf{q}_2$ (previously **q**’)

Consequently, we indeed observed two distinct CDWs that are jointly commensurate with the lattice and remain locked out of equilibrium (Fig. 3 in the revised main text).

We also thank the Reviewer for bringing up the paper *Phys. Rev. Lett.* 93, 106404 (2004) and acknowledge that $NbSe_3$ also hosts jointly commensurate CDWs similar to $EuTe_4$. In $NbSe_3$, the joint commensurability relation between the CDWs with wavevectors $\mathbf{q}_1$ and $\mathbf{q}_2$ leads to their opposite shifts upon application of a current well above the threshold current. This behavior is consistent with the robustness of the joint commensurability upon photoexcitation demonstrated in our work. **We have now included the above discussion in the revised introduction and a new Supplementary Note 9.**

Other comments:

Reference 4 is not complete: B. Q. et al. Large moiré superstructure of stacked incommensurate charge density waves. Nat. Mater. (2025). Given that this paper is based primarily on the recent observation of the coexisting CDW in EuTe4 cited in this reference, it is difficult to accept this paper for publication unless the ref 4 has been published.

Our reply:
Ref. 4 has now been officially published and **we have updated the reference accordingly**.

The structure of the paper is a bit unusual: you treat about UED then XRD, and finally come back to UED again…

Our reply:
We thank the Reviewer for suggesting ways to improve the organization and structure of our manuscript. As suggested by the Reviewer, **we have significantly improved the presentation by grouping the results based on the physical question addressed** : (1) Static momentum space mapping, (2) CDW amplitude and phase fluctuation dynamics, and (3) CDW correlation length and periodicity dynamics. Following this logic, we have minimized frequent switching between different experimental techniques as much as possible, and now present the results in the order of tr-XRD → tr-ARPES → UED. Moreover, **we substantially improved Figs. 1–3 in the main text to facilitate a clearer understanding of the content**. We believe that the restructured version of the manuscript is now more succinct and easier to follow.

References 34, 35 and 36 do not deal with CDW in RTe3 systems but in either YBaCuO or Chromium. Has to be corrected.

Our reply:
We thank the Reviewer for catching this inconsistency. While our intention was to cite more relevant references not limited to $R\mathrm{Te}_3$, **we have removed Refs. 34-36 to avoid any confusion.**

Fig.3D : Is there a color error in the legend?

Our reply:
We thank the Reviewer for pointing out this typo. In the legend of Fig. 3d, the colors assigned to 1 and 4 mJ/cm^2 were inadvertently swapped. **We have corrected this in the revised paper.**

Fig. 3B : The scheme must be changed because this is transverse fluctuations

Our reply:
We note that while the reduction of the correlation length occurs exclusively along the transverse direction, phase fluctuations can in general occur along both the longitudinal and transverse directions, as these are distinct quantities (Lee et al., *Nat. Commun.* 3, 838 (2012)). **We have updated the figure (Fig. 2b in the revised manuscript) as shown in Fig. R4 above.**

Fig. 3E : is it a fit ? From which data ?

Our reply:
As mentioned in the main text, Fig. 3e shows "the temporal evolution of the CDW amplitude extracted from tr-XRD." In addition, as detailed in Supplementary Note 6 (new Supplementary Note 2), Eqs. S10 and S11 are used to fit the tr-XRD data shown in the new Figs. 2c,d of the main text. The resulting fits are then plotted in the new Figs. 2e,f. **We have emphasized this point in the figure caption.**

In conclusion, I personally find the research topic interesting, and I am impressed by the number of experimental techniques used. However, I find the results—though well executed—somewhat disappointing in terms of their physical significance. The interpretation could also be further developed.

Our reply:
We thank the Reviewer for finding the research topic interesting and the range of experimental techniques employed impressive. We believe that, in the revised manuscript, the interpretation of the data has been substantially further developed, together with a clearer discussion of its physical significance.

Reviewer #3 (Remarks to the Author):

In this work, Oh, Su, Ning and coworkers investigate the ultrafast dynamics of the jointly-commensurate charge density wave (JC-CDW) formed in EuTe4 by using static hard X-ray diffraction in conjunction with time-resolved soft X-ray/ electron diffraction and ARPES. This paper builds on an earlier work from the group that demonstrated the existence of the JC-CDW which describes a moiré-like charge modulation, due to the slight wavevector mismatch between the incommensurate CDWs comprising it (Ref. 4 of the main text).

The present paper adds important insight into the non-equilibrium behavior of the accompanying lattice distortion. The key observation is an unusual anisotropic spot broadening, perpendicular to the monolayer CDW wavevector. The authors assign it to a shear-type topological defect and conclude it to be a characteristic feature of the JC-CDW that arises from the wavevector-locking to the underlying lattice, reminiscent of commensurate CDWs. They further extend the discussion to photoinduced CDW amplitude and phase modulations and develop an analysis of first- and second-order satellite spot intensities in tr-XRD to disentangle their dynamics. The approach is validated by cross-checking with tr-ARPES and UED signatures which are directly sensitive to amplitude and phase fluctuations, respectively.

I am convinced by the validity of the results and the balanced conclusions that are drawn from the data. The observation of shear-type topological defect, directly tied to the formation of the JC-CDW is new and of general interest to the broad audience of Nature Communications. While questions regarding the exact formation mechanism of the shear-type defects remain, I generally support publication after the authors have addressed the below comments:

Our reply:
We thank the Reviewer for providing a precise summary and positive assessment of our work.

(1) Optical moiré engineering: The authors motivate their study specifically by the possibility to optically tune moiré potentials by harnessing the susceptibility of charge-ordered states towards photoexcitation. The wording in abstract and conclusions give the reader the impression that optical tunability is a unique property of JC-CDWs and there is no mention of the photoinduced modulation in twist angle that was shown by Duncan et. al. (Nature 647, 619–624 (2025)). The statements in the text should reflect the state of research in this regard.

Our reply:
We thank the Reviewer for suggesting clarification regarding the state of prior research. As the Reviewer noted, optical tunability is not unique to the moiré superstructure formed by the JC-

CDW but also applicable to micron-scale heterobilayers (*Nature* 647, 619–624 (2025)). **We have revised the abstract and introduction accordingly to better contextualize the current state of research on ultrafast optical control of moiré superlattices, with appropriate citation of relevant works.**

(2) Figure 1: In the caption it is mentioned that "I denotes the scattering intensity", yet there is no intensity scale in the figure.

Our reply:

We thank the Reviewer for capturing this oversight. **We have now included the intensity scale in Fig. 1b (as reproduced in Fig. R8).**

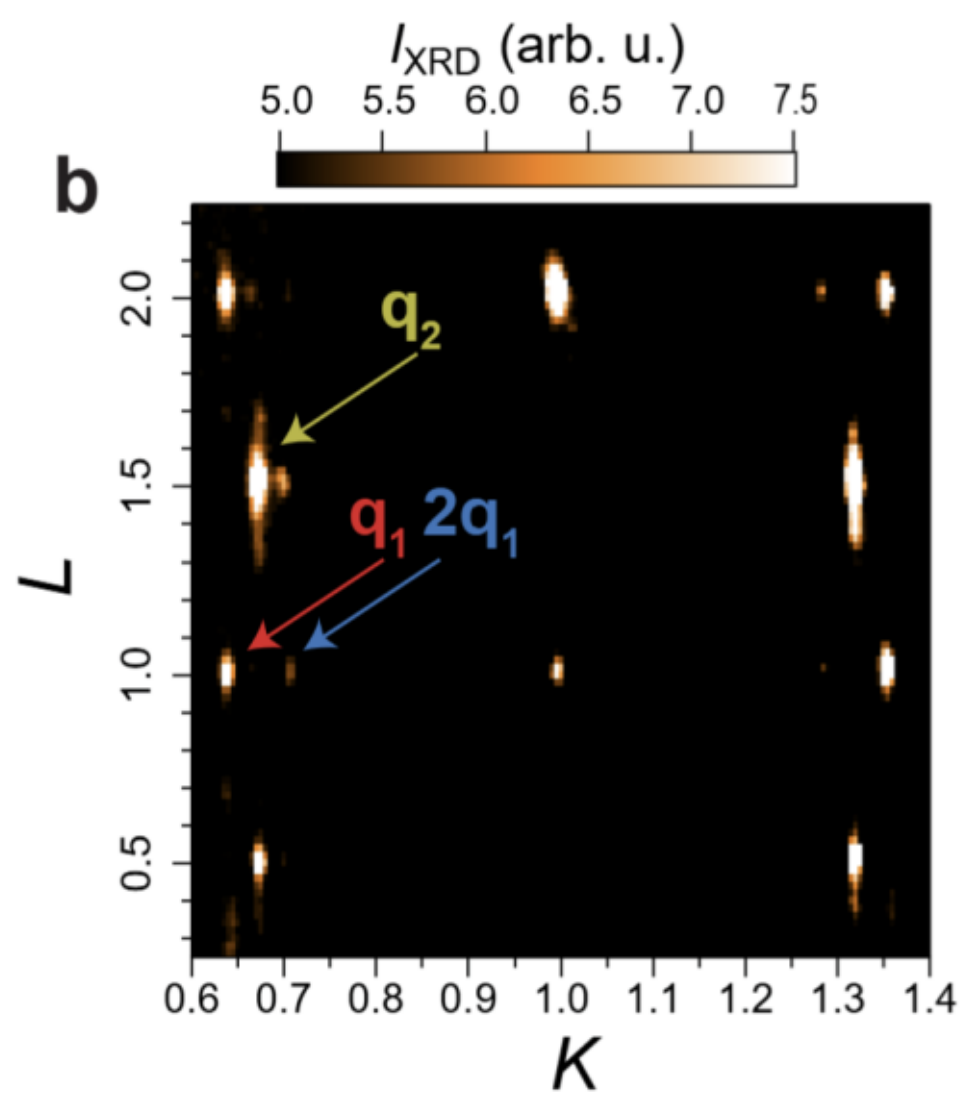

Fig. R8. Equilibrium XRD pattern with the addition of quantitative color scale.

(3) Figure 1: The figure shows an artistic representation of the material structure but the formation of the JC-CDW from I-CDWs in different tellurium planes is not directly obvious. A visual representation of this somewhere in the manuscript would make the concept more accessible to the reader without having to look up Ref. 4.

Our reply:

We thank the referee for the suggestion and agree that a real-space schematic of CDWs in Figure 1 could help the readers better understand the JC-CDW structure. **We have now pictorially** shown

the development of the monolayer and bilayer CDWs on the different Te sheets in Fig. 1c, as reproduced in Fig. R9.

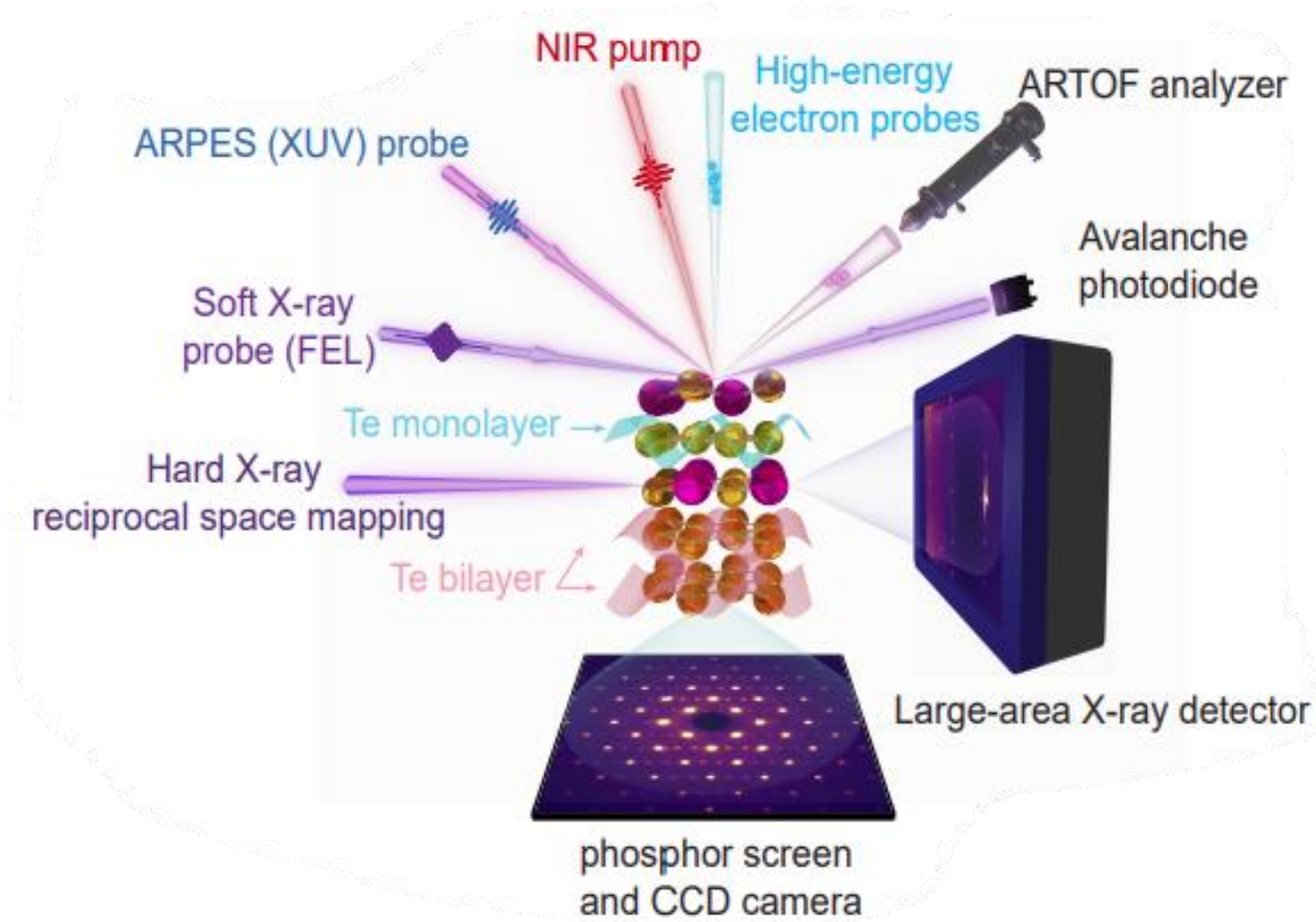

Fig. R9. Schematic of the multi-messenger experimental setup and the lattice structure of $EuTe_4$ updated with schematics of monolayer and bilayer charge density waves

(4) Polarization dependence: As the employed excitation energies in UED and ARPES measurements lie close to the CDW gaps of the material, indicated in Fig. S8, one could expect strongly anisotropic optical properties with respect to the periodic lattice distortion. Similarly, electron-phonon coupling may depend on the excited electronic states. As the polarization is only specified in the XRD methods section, I would like the authors to comment, if they observed a polarization dependence and if they used the same polarization throughout all experiments.

Our reply:

We note that the photon energy of pump excitation (1.2-1.5 eV) employed in UED and tr-ARPES measurements is much higher than the CDW gap energy (~0.6 eV). In this above-gap excitation regime, the CDW melting is induced by incoherent excitation of carriers and should be polarization-insensitive given the near tetragonality of the underlying lattice (Cheng et al., *Nat. Phys.* 20, 54–60 (2024)). To validate this assumption, we have performed new tr-ARPES measurements with pumps of both S- and P- polarizations. As shown in Fig. R10, the in-gap intensity dynamics indeed exhibit negligible pump polarization dependence. Additionally, although the experimental geometries vary across different techniques, we have consistently

employed a S-polarized pump in all measurements in the present work. **We have now clarified this consistency and the insensitivity to pump polarization in the Methods.**

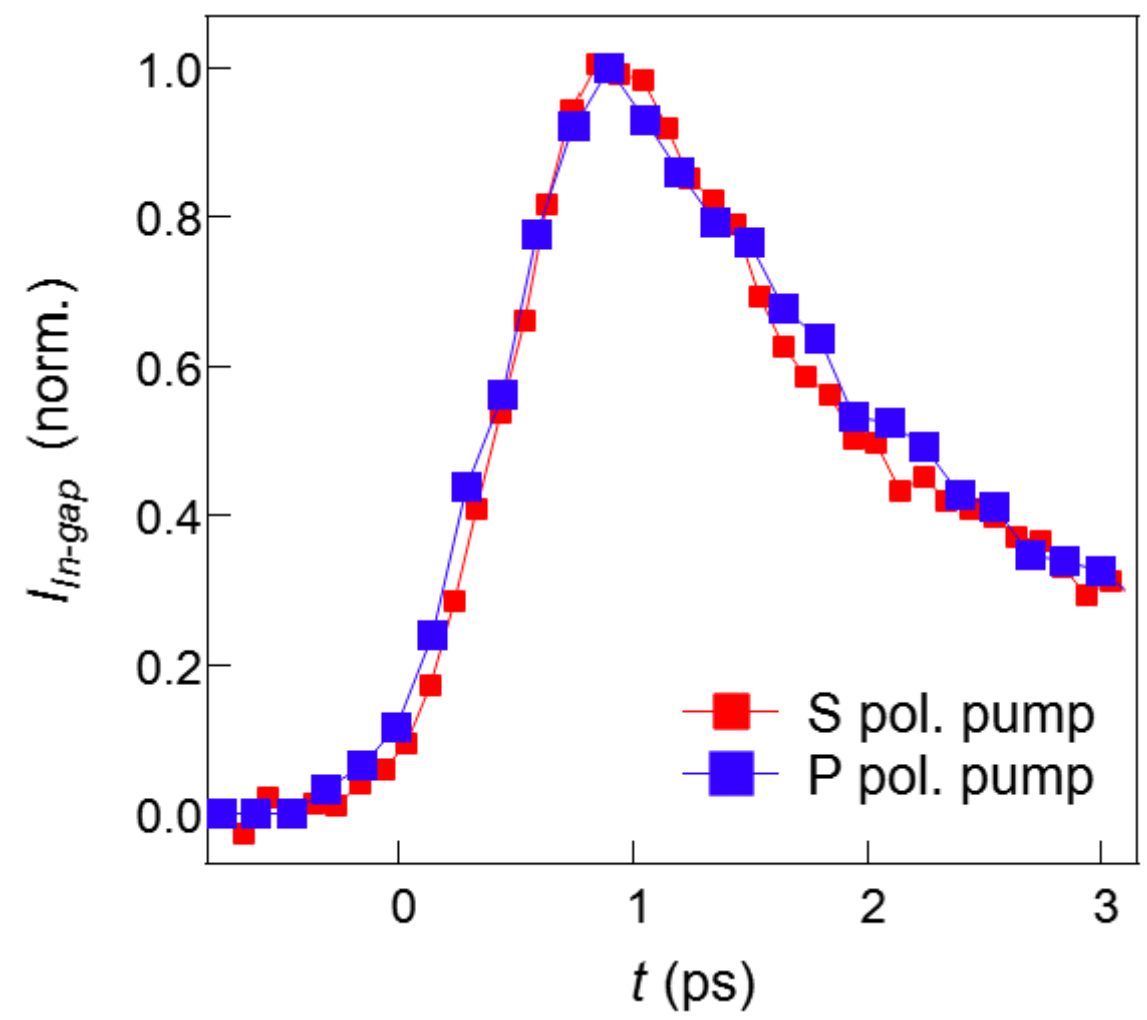

Fig. R10. In-gap intensity dynamics measured by trAPRES with different pump polarizations. There is no discernible dependence on pump polarization.

(5) Bilayer CDW detection in UED: In line 122, it is stated that the bilayer CDW is not detectable in UED experiments. This point is not further commented and thus not directly obvious to a non-expert reader.

Our reply:

The bilayer CDW wavevector is $\mathbf{q}_2$ = (0, 0.678, 0.5) with half-integer, nonzero L. On the other hand, our UED measurements were performed in a transmission geometry, only allowing access to the (H, K, 0) plane. This geometrical constraint hinders the detection of the bilayer CDW satellite peaks. **We have now clearly stated this limitation in the main text for clarity.**
